# Supplementary material for: Notch activity is modulated by the aGPCR Latrophilin binding the DSL ligand in C. elegans
Source: Nat Commun. 2025 Jul 12;16:6461. doi: 10.1038/s41467-025-61730-0 (PMC12255766; doi:10.1038/s41467-025-61730-0)
Supplement: Supplementary file 1 — Supplementary Information [file 41467_2025_61730_MOESM1_ESM.pdf]

# SUPPLEMENTARY INFORMATION

## Supplementary Figures and Figure Legends

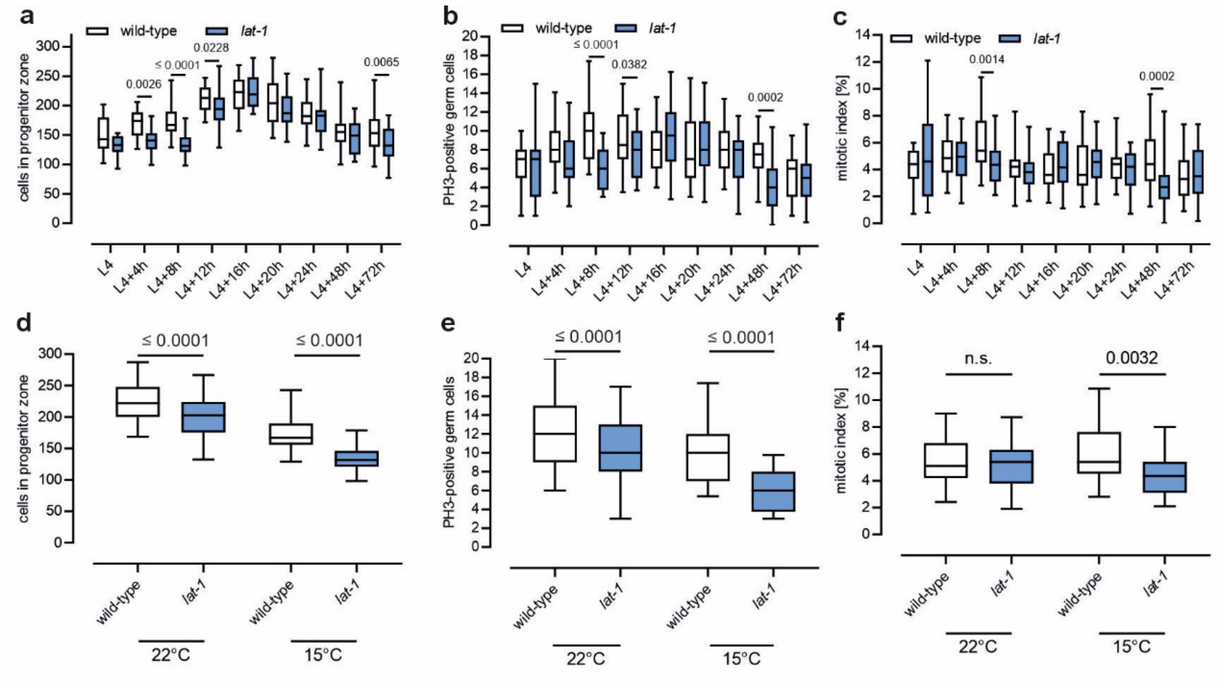


**Supplementary Figure 1. LAT-1 affects germ cell proliferation at specific time points during very early adult stages, an effect that persists at different temperatures.** (a)-(c) Gonads of *lat-1* animals at different ages after L4 at 15°C stained with DAPI and anti-PH3 antibody show differences compared to wild-type controls. (a) In *lat-1* mutant gonads, the size of the progenitor zone is smaller than in wild-type controls at the age of L4 + 4 h to L4 + 12 h as well as L4 + 72 h. (b) Likewise, the number of PH3-positive germ cells at L4 +8 h to L4 + 12 h and L4 + 48 h was significantly decreased in the progenitor zone of *lat-1* hermaphrodites. (c) The mitotic index was significantly altered in *lat-1* mutants only at L4 + 8 h and L4 + 48 h compared to wild-type levels. (d)-(f) The observed alterations in progenitor zone size (d), PH3-positive germ cells (e), but not the mitotic index (f) in *lat-1* gonads at the age of L4 + 8 h persist at 22 °C compared to 15 °C.

Data are based on DAPI- and anti-PH3-stained gonads. n = 22 in 4 independent experiments. Graph raw data and p values are provided in the Source Data. Graph details and statistics are: Box plots with median (center), interquartal range, $5^{th}$ (lower whisker) and $95^{th}$ (upper whisker) percentiles. One-way ANOVA with Bonferroni post-hoc test (a)-(c). Two-sided unpaired t-test without multiple comparison correction (d)-(f).

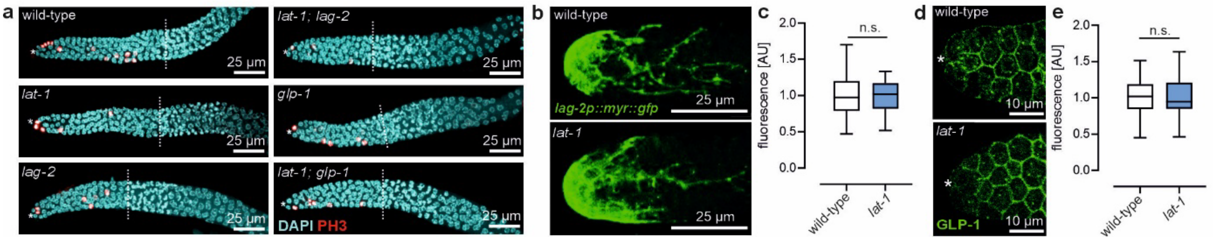


**Supplementary Figure 2. Gonads of *lat-1*, *lag-2*, and *glp-1* single and double mutants and expression of Notch pathway components in *lat-1* gonads.** (a) Representative DAPI- and PH3-stained gonads of L4 + 8 h-old hermaphrodites carrying *lat-1(ok1465)*, *lag-2(q420),* or *glp-1(bn18)* as well as of hermaphrodites harboring the double mutants *lat-1(ok1465); lag-2(q420)* or *lat-1(ok1465); glp-1(bn18)*. These images formed the basis for the quantification of proliferative zone size (Fig. 2d) and PH3-positive germ cell numbers (Fig. 2e). (b), (c) Expression of *lag-2* is not altered in *lat-1* gonads. Representative images of the distal gonads of L4 + 8 h-old hermaphrodites expressing *lag-2p::myr::gfp* (b). These images were used to quantify *lag-2* expression based on fluorescence intensity of the reporter in wild-type and *lat-1* mutants (c). Wild-type: n = 27 in 4 independent experiments, *lat-1*: n = 23 in 4 independent experiments. (d), (e) Expression of *glp-1* in the gonads of *lat-1* hermaphrodites, assessed using the GLP-1 NICD::V5 reporter *glp-1(q1000[glp-1::4xV5])* is not different from that in wild-type individuals (d). Shown are exemplary images of distal gonads. Quantification of these images confirms that total *glp-1* expression does not change in the absence of *lat-1* (e). Note that subcellular distribution of the reporter changes as described in Fig. 2f, g. Wild-type: n = 31 in 6 independent experiments, *lat-1*: n = 28 in at least 4 independent experiments.

Graph raw data and p values are provided in the Source Data. Graph details and statistics are: (c), (e): Box plots with median (center), interquartal range, 5$^{th}$ (lower whisker) and 95$^{th}$ (upper whisker) percentiles. Two-sided unpaired t-test without multiple comparison correction. n.s. = not significant.

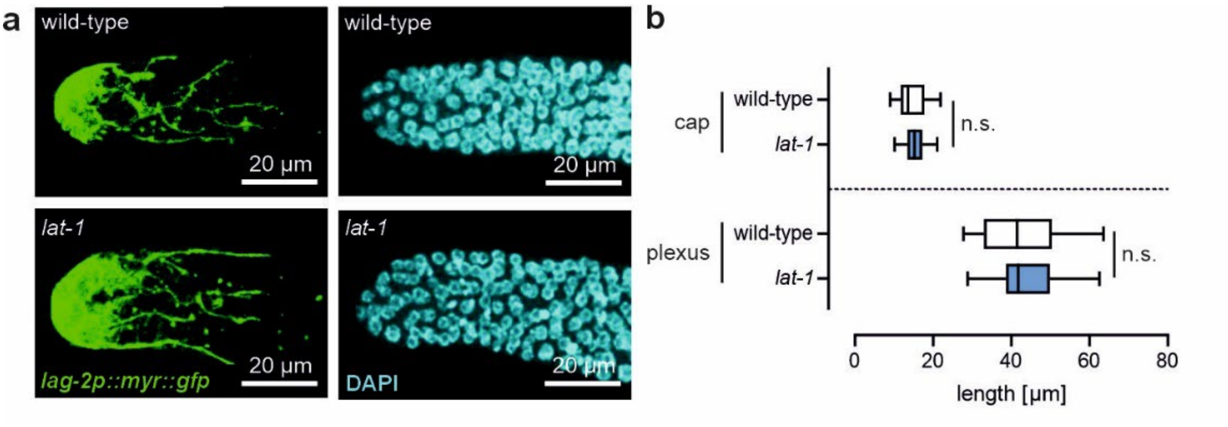


**Supplementary Figure 3. The morphology of the DTC is unaltered in *lat-1* mutants.** (a) Wild-type and *lat-1* mutant DTC visualized by *lag-2p::myr::gfp* expression in 8-h-post-L4 animals. The morphology of the *lat-1* mutant DTC is largely intact compared to the wild-type DTC. The extension of the plexus over the proliferative zone is unchanged. (b) Quantification of images from (a). Cap as well as plexus lengths in *lat-1* gonads are indistinguishable from those in wild-type animals. n.s. = not significant. Cap measurements: wild-type: 28 replicates in 4 independent experiments, *lat-1*: 23 replicates in 3 independent experiments. Plexus: wild-type: 27 replicates in 4 independent experiments, *lat-1*: 22 replicates in 3 independent experiments.

Graph raw data and p values are provided in the Source Data. Graph details and statistics are: (b): Box plots with median (center), interquartal range, 5$^{th}$ (lower whisker) and 95$^{th}$ (upper whisker) percentiles. Two-sided unpaired t-test without multiple comparison correction. n.s. = not significant.

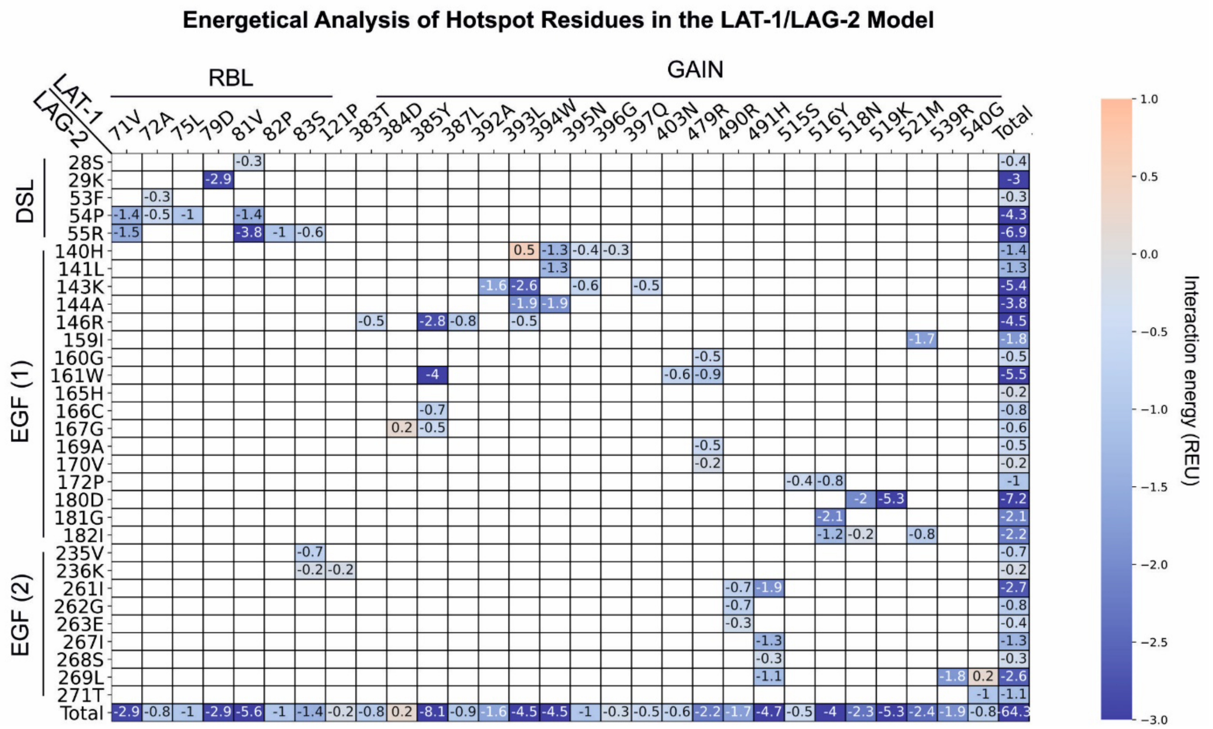


**Supplementary Figure 4. Contact map for the per residue energy contribution of wild-type LAT-1 and LAG-2 interface.** An energy breakdown based on the final energetically minimized Rosetta models of the AlphaFold2 Multimer model shows the average pairwise energetical contribution in Rosetta Energy Units (REU). REU are internal scoring units used by the Rosetta modeling software to estimate the energetic contribution of individual residues to the protein-protein interface. They are not equivalent to kcal/mol and should be interpreted comparatively. More negative values represent stronger stabilizing interactions. A negative number and darker color correlate with stronger energetical contribution between the extracellular regions of LAT-1 and LAG-2. Shown residues were selected by score, with a calculated binding energy higher than +0.2 REU or lower than -0.2 REU.

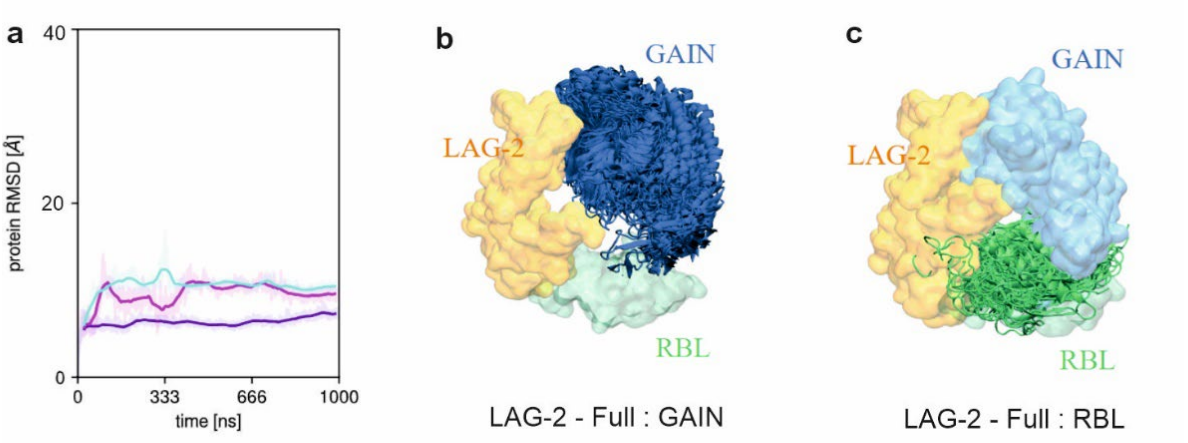


**Supplementary Figure 5. Molecular Dynamics (MD) Simulations of the best ranked LAG-2/LAT-1 complex show stable LAG-2-GAIN interactions.** (a) Root-mean square deviation (RMSD) traces of the simulated complex model. Opaque traces represent moving average over 50 ns for three independent trajectories colored in cyan, magenta and purple, with raw data shown transparent in the background. (b), (c) Flexibility of RBL (green) and GAIN (blue) protein domains relative to LAG-2 (orange) shown in snapshots overlaid with time-dependent conformations every 100 ns shown as cartoon: The full complex trajectory with time-dependent GAIN conformations shows stable interaction regions (b) with a stable relative conformation of the RBL domain in the full complex (c).

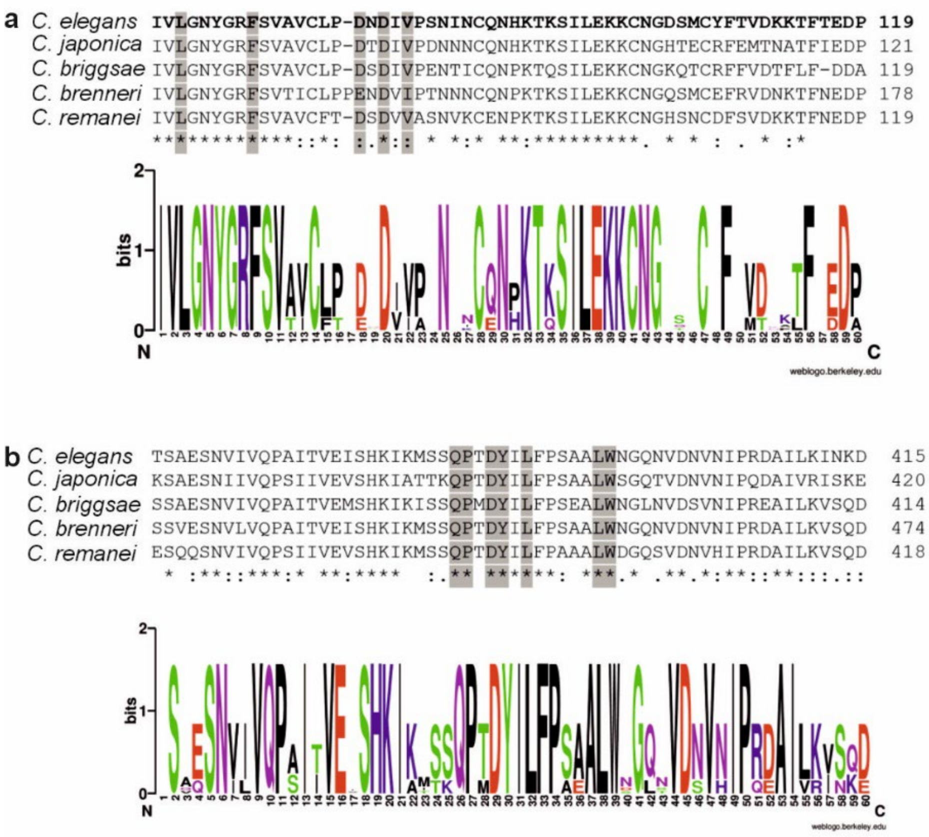


**Supplementary Figure 6. The residues comprising the binding interface of LAT-1 are conserved in *Caenorhabditis* species.** (a) Top: Multiple sequence alignment of related Latrophilin 1 proteins in *Caenorhabditis* species, focusing on the interaction interface of the RBL domain with LAG-2. Residues essential for binding mutated in the course of the study are shaded in grey. Bottom: Sequence logo of the LAT-1 RBL domain shows residue conservation across various nematode species, with highly conserved positions represented by taller letters. (b) Top: Multiple sequence alignment of Latrophilin 1 proteins in *Caenorhabditis* species, focusing on the GAIN interaction interface with LAG-2. Bottom: Sequence logo of the LAT-1 GAIN domain indicates residue conservation across various nematode species, with highly conserved positions represented by taller letters.

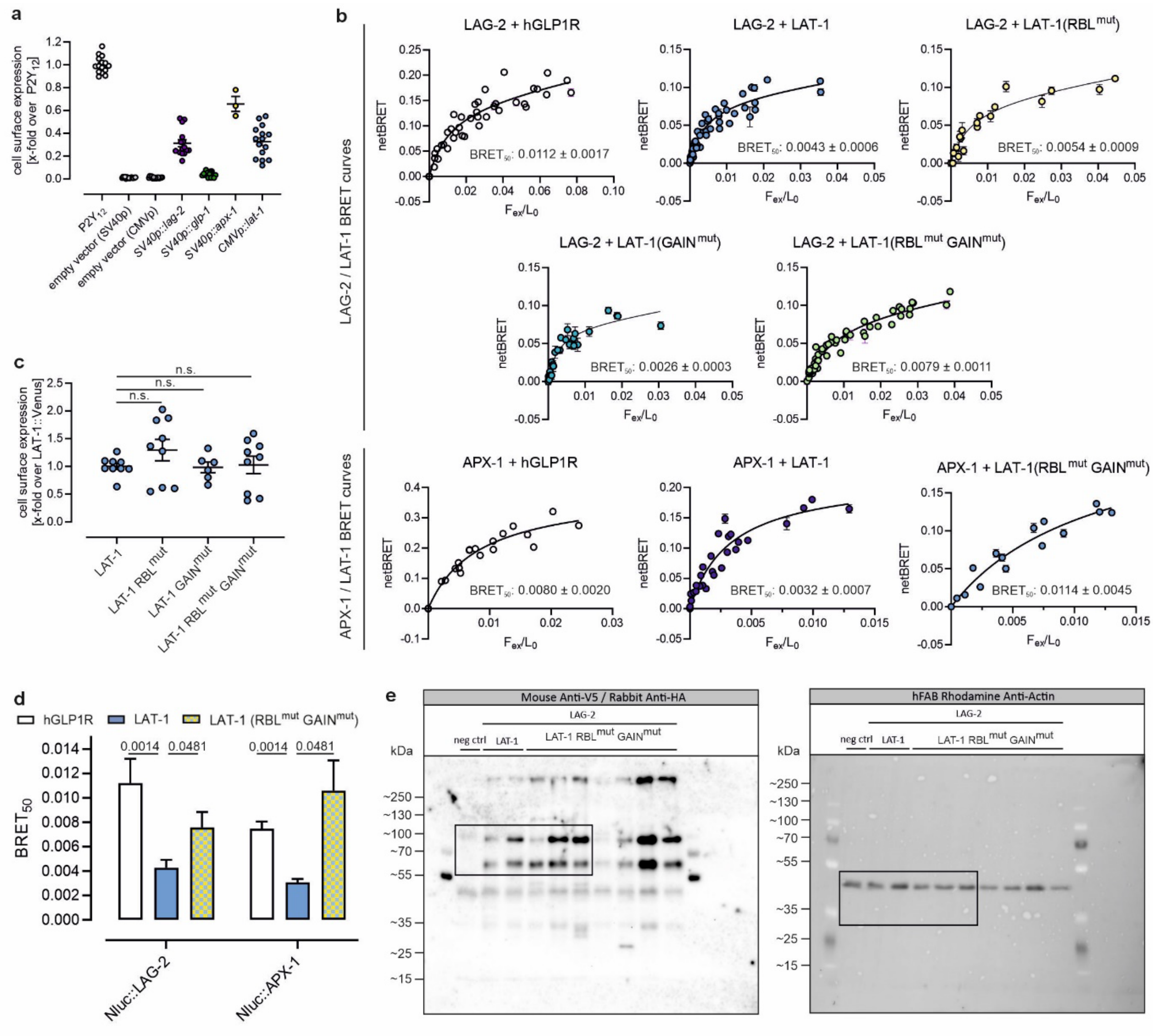

**Supplementary Figure 7. Characterization of NanoBRET and BiFC constructs.** (a) Cell surface expression of *lag-2*, *glp-1*, *apx-1*, and *lat-1* in HEK293 cells by ELISA displayed. Human $P2Y_{12}$ served as positive control. The non-specific OD values are: empty vector (SV40p): 0.0072 ± 0.0042; empty vector (CMVp): 0.0075 ± 0.0040, $P2Y_{12}$: 0.6564 ± 0.1511 (set to 1). *lag-2*, *glp-1*, and *apx-1* are under the control of the SV40 promoter and *lat-1* is CMV promoter-driven. Number of independent experiments (all in triplicates): *SV40p::apx-1*: 3 independent experiments; all other samples: 5 independent experiments. (b) Plots displaying netBRET values from the NanoBRET analyses of LAT-1 (LAT-1::Venus) and respective constructs carrying point mutations in RBL and/or GAIN domain with LAG-2 (Nluc::LAG-2) or APX-1 (Nluc::APX-1) in HEK293 cells over increasing amounts of transfected acceptor plasmid. The human hGLP1R::Venus served as negative control. Note that LAG-2 + hGLP1R shows a higher $netBRET_{max}$ value due to the different orientation of this complex. Different $BRET_{50}$ values reflect different protein-protein affinities. $BRET_{50}$ values are stated for each donor:acceptor pair. All experiments were performed in technical quadruplicate. Independent replicates: LAG-2 + hGLP1R: 8, LAG-2 + LAT-1: 11, LAG-2 + LAT-1($RBL^{mut}$): 4, LAG-2 + LAT-1($GAIN^{mut}$): 6, LAG-2 + LAT-1($RBL^{mut}$ $GAIN^{mut}$): 5, APX-1 + hGLP1R: 4, APX-1 + LAT-1: 6, APX-1 + LAT-1($RBL^{mut}$ $GAIN^{mut}$): 3. (c) ELISA reveals an unaltered cell surface expression of *lat-1($RBL^{mut}$ $GAIN^{mut}$)::Venus* mutants compared to unmutated LAT-1 in HEK293 cells. 3 independent experiments for all plasmids, except *Venus::lat-1 $GAIN^{mut}$* (2 independent replicates), all performed in triplicate. (d) APX-1 and LAT-1 interact *in vitro.* NanoBRET assays indicate a similar affinity of APX-1 for LAT-1 as LAG-2. Introducing point mutations in RBL and GAIN domain reduces the affinity in a comparable manner to LAG-2 and LAT-1, indicating that

the same residues in LAT-1 are required to mediate the interaction. Number of independent replicates (each in quadruplicate): LAG-2 + hGLP1R: 8, LAG-2 + LAT-1: 11, LAG-2 + LAT-1($RBL^{mut}$ $GAIN^{mut}$): 10, APX-1 + hGLP1R: 4, APX-1 + LAT-1: 6, APX-1 + LAT-1($RBL^{mut}$ $GAIN^{mut}$): 3. Corresponding netBRET values and curves are shown in (b). (e) Uncropped Western blots (related to Fig. 4d). Left: LAT-1 (V5-tagged, 81 kDa (autocatalytically cleaved)), LAG-2 (HA-tagged, 57 kDa), and mutated LAT-1($RBL^{mut}$ $GAIN^{mut}$) (V5-tagged, 81 kDa (autocatalytically cleaved)). Right: reference protein actin (~42 kDa). Boxes indicate crops displayed in Fig. 4d. Western Blot was performed twice with 60-80 worms per sample.

Graph raw data and p values are provided in the Source Data. Graph details and statistics are: (a), (c), (d): Mean ± SEM. One-way ANOVA with Bonferroni post-hoc test. n.s. = not significant.

# Supplementary Tables and Table legends

## Supplementary Table 1. Reagents and resources

| REAGENT or RESOURCE | SOURCE | IDENTIFIER |
|---|---|---|
| **Bacterial and Virus Strains** | | |
| *E. coli* DH5α | ThermoFisher Scientific | 18258012 |
| *E. coli* OP50 | CGC | N/A |
| **Primary antibodies** | | |
| Rabbit anti-phospho histone H3 (Ser10) | Merck Millipore | 06-570 |
| Mouse anti-V5 SV5-Pk1 | BioRad | MCA1360 |
| Anti-HA-peroxidase, High Affinity | Sigma | 54193500 |
| **Secondary antibodies** | | |
| Goat anti-Rabbit IRDye 680RD-conjugated | LiCor | 926-68071 |
| Goat anti-Mouse IgG (H+L), F(ab')2 Fragment CF 568 | Biotium | 20109 |
| Anti-rabbit IgG, HRP-linked Antibody | Cell Signaling Technology | 7074 |
| Goat Anti-Mouse IgG (H+L)-HRP Conjugate | BioRad | 1721011 |
| anti-Actin hFAB-Rhodamine | BioRad | 12004164 |
| **Chemicals** | | |
| 4,6 diamidine-2-phenylindole (DAPI) | Sigma | D9542 |
| Fluoromount G | ThermoFisher Scientific | 00-4958-02 |
| Levamisole hydrochloride | BLDpharm | MFCD00012675 |
| **Enzymes** | | |
| Alt-R S.p. HiFi Cas9 Nuclease V3, 100 µg | Integrated DNA Technologies, Inc | 1081060 |
| **Critical Commercial Assays** | | |
| Click-iT EdU Alexa Fluor 488 Imaging Kit | ThermoFisher Scientific | C10337 |
| Click-iT EdU Alexa Fluor 647 Imaging Kit | ThermoFisher Scientific | C10340 |
| NEBuilder HiFi DNA Assembly | New England Biolabs | E2621L |
| Zero Blunt TOPO PCR Cloning Kit | ThermoFisher Scientific | 450245 |
| **Experimental Models: Cell Lines** | | |
| HEK293 | German Collection of Microorganisms and Cell Cultures (DSMZ) | ACC 305 |
| HEK293T | German Collection of Microorganisms and Cell Cultures (DSMZ) | ACC 635 |
| **Experimental Models: Organisms/Strains** | | |
| *Caenorhabditis elegans* strains, see *C. elegans* maintenance and strains and Table S3 | This paper | N/A |
| **Oligonucleotides** | | |
| See Supplementary Table 1 | Sigma-Aldrich, IDT, Microsynth | N/A |
| **Scientific instruments** | | |
| TCS SP8 STED 3X | Leica | |
| Spark plate reader | Tecan | |

**Supplementary Table 2. Oligonucleotides used in this study**

| Primer | Sequence (5' -> 3') |
|---|---|
| Bifc_1F | CACAACATCGAGAGTTTAAACTAAGCGGCCGCAG |
| Bifc_2R | CTGCGGCCGCTTAGTTTAAACTCTCGATGTTGTG |
| Bifc_3Fp | GAATTCATCTCGAGATGGTGAGCAAGGGCGAGGAGCTGTTCACC |
| Bifc_4Rp | GGTGGGCCCGCGGGTACAATTGCTAGCCAA |
| Bifc_5F | AGGTTTAAACGCCGCCGCAGCAGCCAGCAACAAGCCGACAACGGATGAAAGTGGA |
| lat-1_881F | GCCAATCTTTCCCTACTCTCCTCT |
| lat-1_1467Fp | GGCGAAAGTTGGAGTAACAGTCAG |
| lat-1_1468Rp | GCATGTACTGCCAGGACCTGGATTACAAGGATGACGACGATAAGTAAG |
| lat-1_1469Fp | GCTCCATGGATGCGGCCGCAGGATCCAGACA |
| lat-1_1470Rp | CGCTGCGGCAGCCTTGTACAGCTCGTCCATGC |
| lat-1_1473Fp | ACGGTACCTCACTTATCATCGTCGTCCTTGT |
| lat-1_1474Rp | TGAGGTACCGAGCTCGAATTCA |
| lat-1_1481F | CTCGAGATGATCGCTTACTTCCTCTT |
| lat-1_1482R | TCTCGAGCAGAAGAACGGCATCAAGGC |
| lat-1_1483Fp | ACCTGCCTCCCAGTCCTCCAAGCCCAGAAGAACGGCATCAAGGC |
| lat-1_1483Fp | TCGGTGGTAGGCTTGTTGCTGGCGTAGTCGGGGACGTCGT |
| lat-1_1484Rp | GAGGAGTAAGAGGAAGTAAGCGATCATCTCGAGATGAATTCGGTGGG |
| lat-1_1484Rp | ACGACGTCCCCGACTACGCCAGCAACAAGCCTACCACCGA |
| lat-1_1694Fp | TCGTCGTCATCCTTGTAATCCAGGTCCTGGCAGTACATGC |
| lat-1_1695Rp | TGAACGGCAGCTTCTACTGCGATTACAAGGATGACGACGATAAG |
| lat-1_1695Rp | GCAATTGCATTCCCGTCAGCCGCTTTATG |
| lat-1_1700F | TGCTGTTGCCTGTGACGACATCTTTATTTTGTGTG |
| lat-1_1703R | TGGCGTCAGGCGGTGGAGGTCAAACATCTAGTAATGAGCAGCTC |
| lat-1_1708F | TCTCCGCCCATCAGTTGGCTGGCGTAGTCGGGGACGTCGT |
| lat-1_1709R | GCGTTTTTTGCGCCAATCGCCCAGAAGAACGGCATCAAGGC |
| lat-1_1709R | ACGACGTCCCCGACTACGCCAGCCAACTGATGGGCGGAGA |
| lat-1_1712F | TGAATTCGGTGGGCCCGCGGGTACAAT |
| lat-1_1713R | GCTCCATGGATGCGGCCGCAGGATCCAGACA |
| lat-1_1714F | CGCTGCGGCAGCCTTGTACAGCTCGTCCATGC |
| lat-1_1731Fp | GCCTCCCAGTCCTCCAAGCCGACTACAAAGACGATGACGACAAGGCCGC |
| lat-1_1732Rp | TCGTCATCGTCTTTGTAGTCGGCTTGGAGGACTGGGAG |
| lat-1_1819F | TCGTCGTCATCCTTGTAATCGCAGTAGAAGCTGCCGTTCA |
| lat-1_1822R | ACGTCGTAGGGGTAGCTGGCGATAGGGGCGAAG |
| lat-1_1852R | GCTTTCTTCGCCCCTATCGCCTACCCCTACGACGTCCCCGAC |
| lat-1_1853F | CAGCAGGATCAGCAGCACCCGCATGGTGGCGGTACCAGATCTCCCG |
| lat-1_1869Fp | CCCCGACTACGCCCAACTGATGGGCGGAGAGTG |
| lat-1_1870Rp | GCCCGGGGCCACCATGATCGCCTACTTCCTGCT |
| lat-1_1927Fp | GAGTAAAATTAGAAGAACTCGCATCTCGAGATGAATTCGGTGGG |
| lat-1_1928Rp | CTCGAGATGATCGCTTACTTCCTCTT |
| lat-1_2006Rp | TCTATATCACCGCCGACAAGCAGAAGAACGGCATCAAGGC |
| lat-1_2009Fp | GCCTTGATGCCGTTCTTCTGCTTGTCGGCGGTGATATAGACGTT |
| lat-1_2036Rp | GAGGTTCTGGTGGCGGAGGTTCTAGCAACAAGCCGACAACGGA |
| lat-1_2167F | CGTGATTACAAGGATGACGATGACAAGAGACCTCGCCGTTTCACATACCTAC |
| lat-1_2168R | TTCACACAGGAAACAGCTATGACCATTTATCCAGTTGAACTATTTACTCCAGGTGAC |
| lat-1_2169F | CATCGATGCTCCTGAGGCTCCCGATGCTCCATCTGATGGGAAAACTTCAGTGAGC |

| | |
|---|---|
| lat-1_2170R | CGTGATTACAAGGATGACGATGACAAGAGACCTCGCCGTTTCACATACCTAC |
| lat-1_2175F | TTCACACAGGAAACAGCTATGACCATTTATCCAGTTGAACTATTTACTCCAGGTGAC |
| lat-1_2176R | TCTTGGTGAAACGGCGAGGATCTGA |
| lat-1_2177F | AAACTCAGATCCTCGCCGTTTCACC |
| lat-1_2178R | CACGCTGGCTCTTCCGTAGTTGCCCGCCACGATGCTGATCACTTTGC |
| lat-1_2179F | GCTGTCTGCCTGCCCGCTAACGCCATCGCGCCAAGCAACATCAACTGCCAG |
| lat-1_2180R | GACAGAGGCTCTTCCATAGTTTCCTGCGACAATCGAGATTACTTTTCCAGC |
| lat-1_2240R | GACAGAGGCTCTTCCATAGTTTCCTGCGACAATCGAGATTACTTTTCCAGC |
| lat-1_2241F | GCTGTTTGCCTTCCTGCCAATGCCATCGCTCCATCCAACATCAACTGCCAAAAC |
| lat-1_2284F | GCTGTTTGCCTTCCTGCCAATGCCATCGCTCCATCCAACATCAACTGCCAAAAC |
| lat-1_2285R | CTTGCCAGGCCTCCTGCAGCTAATCTAGAGGGCCCGTTTAAACCC |
| lat-1_2402F | TCGGGGACGTCGTAGGGGTACCTGCCCACCATCCCGAG |
| lat-1_2403R | TGCTCGGGATGGTGGGCAGGTACCCCTACGACGTCCCCG |
| lat-1_2491F | TGCAGCGGCTGACGGGAATGCAA |
| lat-1_2492R | TTTCCCTCTGCCGCCGCAGCTAACGGCCAAAATGTCGATAATG |
| lat-1_2499F | TAAACGGGCCCTCTAGATTAGCTGCAGGAGGCCTGGCAAGTGG |
| lat-1_2500R | TCCAGATTACGCTGCCGCCGCAGCAGCCCGAG |
| lat-1_2501F | CCCTGGGGGCGGGGGCCGGCTGAACCTCCTCCACCGGAGT |
| lat-1_2502R | ACTCCGGTGGAGGAGGTTCAGCCGGCCCCCGCCCCCAG |
| lat-1_2503F | ACATCGTATGGGTAGGCTTGGAGGACTGGGAGGC |
| lat-1_2504R | CTCCTCGGACTTGATAGCACCGCCGCCGCAGCAGCCATG |
| lat-1_2505F | TGGGTTAGGGATAGGCTTACCCTCGAGGGCGAAAGTTGGAGTAAC |
| lat-1_2506R | GGGGTCTGGTGAGCGGTGAGAAGGGCGAATTCTGCAGATA |
| lat-1_2511F | TGCGATAGCAGCGGTTGCTGCGCTAGACATCTTGATCTTGTG |
| lat-1_2512R | ACGTTGTAAAACGACGGCCAGTCGCCGGCAGTCGTTTGCCAGGGCTGTTG |
| lat-1_2513F | AGACTTTTGGACCGTGCGCCAAGGGCGAATTCCAGCACAC |
| lat-1_2514R | GTGTGCTGGAATTCGCCCTTGGCGCACGGTCCAAAAGTCT |
| lat-1_2527F | AGTTTAAACGCCGCCGCAGC |
| lat-1_2528R | CTCGATGTTGTGGCGGATCTTGAA |
| lat-1_2529F | GCGGCCGCCTAGACATAGTGACAGGCTG |
| lat-1_2529F | CGAGTAGAAGTCCATCAAGAGTTCAT |
| lat-1_2530R | ACCTGCCTCCCAGTCCTCCAAGCCCAGAAGAACGGCATCAAGGC |
| lat-1_2530R | GGCTTGGAGGACTGGGAG |
| lat-1_2531F | TCGTCGAGTGTAAGAGTTTCTCTCTGTCTGAAACATTCAATTGATTATC |
| lat-1_2532R | AAACTCAGATCCTCGCCGTTTCACC |
| lat-1_2616R | GGGGTCTGGTGAGCGGTGAGAAGGGCGAATTCTGCAGATA |
| lat-1_2617F | AGACTTTTGGACCGTGCGCCAAGGGCGAATTCCAGCACAC |
| lat-1_2618R | ACGACGTCCCCGACTACGCCATGGTGAGCAAGGGCGAGGA |
| lat-1_2618R | GCGAATGGGCAGAATGTTGATAATGTAAATATTC |
| lat-1_2619F | TCCTCGCCCTTGCTCACCATGGCGTAGTCGGGGACGTCGT |
| lat-1_2619F | GGTAGGCTTGTTGCTTGAACCTCCTCCACCGGAGTCGAGCGCTCGCCGAG |
| lat-1_2628F | CCAACTTTTCTTCTTTGCAGTTTGAATCCGAAAAAGTCTC |
| lat-1_2629R | TCGTCGAGTGTAAGAGTTTCAAAAGGCAAATTTGAAAAGTG |
| lat-1_2630F | GAGACTTTTTCGGATTCAAACTGCAAAGAAGAAAAGTTGGAAAAATAGAG |
| lat-1_2631R | CCAACTTTTCTTCTTTGCAGTTTGAATCCGAAAAAGTCTC |
| lat-1_2632F | GTGTGCTGGAATTCGCCCTTGGCGCACGGTCCAAAAGTCT |
| lat-1_2633R | GAGACTTTTTCGGATTCAAACTGCAAAGAAGAAAAGTTGGAAAAATAGAG |

| | |
|---|---|
| lat-1_2652F | ACTGAGCCTCCGCCACCACTATTAGGCTGAGAGGGGTGGA |
| lat-1_2653R | TCCACCCCTCTCAGCCTAATAGTGGTGGCGGAGGCTCAGT |
| lat-1_2654F | TGCTCATTACTAGATGTTTGACCTCCACCGCCTGACGCCA |
| lat-1_2655R | GCTCGACTCCGGTGGAGGAGGTTCAAGCAACAAGCCTACCACCGATG |
| lat-1_2691F | GCGTAATCTGGAACATCGTATGGGTAGGCGATTGGCGCAAA |
| lat-1_2691F | TTGAATGTTTCAGACAGAGAGAAACTCTTACACTCGACGA |
| lat-1_2692R | GATCCATGGAGTCAACTTATGGGTGGA |
| lat-1_2692R | GAAAATGTATTTTCTTGCAGCTGCAAAGAAGAAAAGTTGG |
| lat-1_2693F | GAGGAGTAAGAGGAAGTAAGCGATCATCTCGAGATGAATTCGGTGGG |
| lat-1_2693F | ACTTTTCAAATTTGCCTTTTGAAACTCTTACACTCGACGAACA |
| lat-1_2694R | TGCCGCCGCAGCAGCCCAGAAGAACGGCATC |
| lat-1_2694R | TATCTGCAGAATTCGCCCTTCTCACCGCTCACCAGACCCC |
| lat-1_2695F | ACCTGCCTCCCAGTCCTCCAAGCCCAGAAGAACGGCATCAAGGC |
| lat-1_2695F | TCGTCGAGTGTAAGAGTTTCAAAAGGCAAATTTGAAAAGTG |
| lat-1_2696R | GAGGAGTAAGAGGAAGTAAGCGATCATCTCGAGATGAATTCGGTGGG |
| lat-1_2696R | ACTTTTCAAATTTGCCTTTTGAAACTCTTACACTCGACGAACA |
| lat-1_2697F | TGCCGCCGCAGCAGCCCAGAAGAACGGCATC |
| lat-1_2697F | TATCTGCAGAATTCGCCCTTCTCACCGCTCACCAGACCCC |
| lat-1_2698R | GCGTAATCTGGAACATCGTATGGGTAGGCGATTGGCGCAAA |
| lat-1_2698R | TTGAATGTTTCAGACAGAGAGAAACTCTTACACTCGACGA |
| lat-1_2708F | GATGCGGCCGCCTAGACATAGTGACAGGCTG |
| lat-1_2708F | CCAACTTTTCTTCTTTGCAGCTGCAAGAAAATACATTTTC |
| lat-1_2709R | GATGCGGCCGCCTAGACATAGTGACAGGCTG |
| lat-1_2709R | TCGTCGAGTGTAAGAGTTTCTCTCTGTCTGAAACATTCAATTGATTATC |
| lat-1_2710F | GATGCGGCCGCTTAACAGTAGAAGGAGCCAT |
| lat-1_2710F | GAAAATGTATTTTCTTGCAGCTGCAAAGAAGAAAAGTTGG |
| lat-1_2711R | GATGCGGCCGCATCGAGTAGAAGTCCATCAAGA |
| *p indicates 5' phosphorylation* | |
| **ssODN** | **Sequence** |
| ssODN 1 | TAAACTCAGTTATCATAATAAGACAATGACGTCATGTGACTGTACTGCTCTCACACACTTCGCAGTACTGATGGATGTACGAGGACACGATCTGAATGAAATCGACCAAACGCTTCTCAC |
| ssODN 4 | ATTGTTTCCTGCATTATTCAAATTCGTTGCTGCCGTTTGTTTGTCGATATGGAAGATAAATACCGTTCTACTTTTGTTACAGAACTTTTTTATTGTTAAAAAGTTTAATATAATTGCTC |
| **crRNA** | **Sequence** |
| crRNA 2 | UUCCAACUUUUCUUCUUUGC |
| crRNA 5 | ACAAAAGUAGAACGGUAAGA |
| crRNA 6 | UCGAUAUGGAAGAUAAACAA |
| **tracrRNA** | **Sequence** |
| Alt-R CRISPR-Cas9 tracrRNA | N/A |

**Supplementary Table 3. Plasmids generated in this study**

| Plasmid | Open reading frame | Used for | Worm strain |
|---|---|---|---|
| pNF001 | *hsp-16.41p::HA::VC155::ala* [*Nco*I and *Not*I restriction sites] | Subcloning | n.a. |
| pNF002 | *hsp-16.41p::HA::VC155::ala* [*Xho*I and *Eco*RI restriction sites] | Subcloning | n.a. |
| pNF017 | *hsp-16.41p::lag-2sp::FLAG::ala::lag-2(1-193)::VC155::lag-2(194-401)* | BiFC analysis | APR771, APR834, APR841, APR842 |
| pSP188 | *hsp16.41p::lat-1sp::FLAG::VN173::ala::lat-1* | BiFC analysis | APR751 |
| pSP228 | *hsp16.41p::lag-2sp::VN173::lag-2* | BiFC analysis | APR739 |
| pSP229 | *hsp16.41p::glp-1sp::VC155::glp-1* | BiFC analysis | APR751 |
| pSP234 | *hsp16.41p::lag-2* | Competition BiFC | APR834, APR841, APR842 |
| pSP246 | *hsp16.41p::lat-1sp::FLAG::VN173::gly-ser::lat-1* | BiFC analysis | APR771, APR834, APR841, APR842 |
| pSP314 | *SV40p::glp-1sp::HA::glp-1::FLAG* | Surface ELISA | n.a. |
| pSP318 | *SV40p::lag-2sp::HA::lag-2::FLAG* | Surface ELISA | n.a. |
| pSP334 | *CMVp::lphn-1sp::HA::lat-1::FLAG* | Surface ELISA | n.a. |
| pSP359 | *SV40p::lag-2sp::HA::lag-2(18-193)::gly-ser::Nluc::gly-ser::lag-2(194-401)* | BRET | n.a. |
| pSP360 | *CMVp::lphn-1sp::HA::Venus::lat-1::FLAG* | BRET | n.a. |
| pSP371 | *CMVp::lphn-1sp::HA::Venus::lat-1[GAIN$^{mut}$]::FLAG* | BRET | n.a. |
| pSP392 | *CMVp::lphn-1sp::HA::Venus::lat-1[RBL$^{mut}$ GAIN$^{mut}$]::FLAG* | BRET | n.a. |
| pSP395 | *CMVp::lphn-1sp::HA::Venus::lat-1[RBL$^{mut}$]::FLAG* | BRET | n.a. |
| pSP396 | *CMVp::GLP1Rsp::HA::Venus::GLP1R* | BRET | n.a. |
| pSP403 | *hsp-16.41p::lag-2sp::HA::ala::lag-2(1-193)::vc155::lag-2(194-401)* | BiFC analysis | APR920, APR974 |
| pSP404 | *hsp16.41p::lat-1sp::V5::VN173::lat-1* | BiFC analysis | APR920 |
| pSP406 | *hsp16.41p::lat-1sp::V5::VN173::lat-1[RBL$^{mut}$ GAIN$^{mut}$]* | BiFC analysis | APR974 |
| pSP427 | *CMVp::lag-2sp::HA::apx-1(21-191)::gly-ser::Nluc::gly-ser::(192-515)* | BRET | n.a. |

**Supplementary Table 4. *C. elegans* strains used in this study**

| Strain | Genotype | Used for | Source | Reference |
|---|---|---|---|---|
| Bristol N2 | N/A | Phenotyping assays (PH3, EdU), Western blot | CGC | 11 |
| APR873 | *lat-1(ok1465) II* | Phenotyping assays (PH3, EdU), Western blot, anus phenotyping, octanol assays | CGC | |
| WD881 | *on172(rec-8::mNeonGreen) IV* | REC-8 analysis | Brent Derry | 9 |
| APR886 | *lat-1(ok1465) II; on172(rec-8::mNeonGreen)IV* | REC-8 analysis | This paper | |
| JH2874 | *pgl-1(ct131) him-3(e1147) III; ozIs5 [gld-1::GFP + unc-119(+)]* | GLD-1 analysis | CGC | 12 |
| APR756 | *pgl-1(ct131) him-3(e1147) III; ozIs5 [gld-1::GFP + unc-119(+)] (outcrossed 4x)* | GLD-1 analysis | This paper | |
| JK1277 | *lag-2(q420) V* | Epistasis analysis, anus phenotyping, octanol assays | CGC | 13 |
| APR569 | *lat-1(ok1465) II; lag-2(q420) V* | Epistasis analysis, anus phenotyping, octanol assays | This paper | |
| HA1019 | *osm-11(rt142) X.* | Octanol assays | | 14 |
| DG2389 | *glp-1(bn18) III* | Epistasis analysis | CGC | 15 |
| APR768 | *lat-1(ok1465) II; glp-1(bn18) III* | Epistasis analysis | This paper | |
| JK5933 | *glp-1(q1000[glp-1::4xV5]) III* | Notch activation analysis, *glp-1* expression quantification | CGC | 16 |
| APR778 | *lat-1(ok1465) II ; glp-1(q1000[glp-1::4xV5]) III* | Notch activation analysis, *glp-1* expression quantification | This paper | |
| APR868 | *lat-1(apr1[lat-1[1-581]::GFP^HygR]) II; glp-1(q1000[glp-1::4xV5]) III* | Notch activation analysis | This paper | |
| APR862 | *glp-1(q1000[glp-1::4xV5]) III; lag-2(q420) V* | Notch activation analysis | This paper | |
| JK4475 | *qIs153 [lag-2p::MYR::GFP + ttx-3p::DsRed] V.* | DTC morphology, *lag-2* expression quantification | CGC | 17 |
| APR524 | *lat-1(ok1465) II, qIs153 [lag-2p::MYR::GFP + ttx-3p::DsRed] V.* | DTC morphology, *lag-2* expression quantification | This paper | |
| APR739 | *aprEX301 [pSP228 pSP229 pRF4]* | BiFC analysis, pSP228 and pSP229 injected at 3 ng/µL | This paper | |
| APR751 | *aprEX304 [pSP188 pSP229 pRF4]* | BiFC analysis, pSP188 and pSP229 injected at 1 ng/µL | This paper | |
| APR771 | *aprEx309[pSP246 pNF17 pRF4]* | BiFC analysis, pSP246 and pNF017 injected at 1 ng/µL | This paper | |

| | | | | |
|---|---|---|---|---|
| APR834 | *aprEx309[pSP246 pNF17 pRF4]; aprEX341[pSP234 [50 ng/µL] IR98 pBlueScript SKII]* | Competition BiFC, pSP234 injected at 50 ng/µL | This paper | |
| APR841 | *aprEx309[pSP246 pNF17 pRF4], aprEx342[pSP234 [5 ng/uL], IR98 pBlueScript SKII]* | Competition BiFC, pSP234 injected at 5 ng/µL | This paper | |
| APR842 | *aprEx309[pSP246 pNF17 pRF4], aprEx343[pSP234 [10 ng/uL], IR98 pBlueScript SKII]* | Competition BiFC, pSP234 injected at 10 ng/µL | This paper | |
| APR920 | *aprEx370[pSP403 pSP404 pRF4]* | BiFC analysis, pSP403 and pSP404 injected at 15 ng/µL | This paper | |
| APR974 | *aprEx384[pSP406 pSP403 pRF4]* | BiFC analysis, pSP406 and pSP404 injected at 15 ng/µL | This paper | |
| APR742 | *lat-1(apr1[lat-1[1-581]::GFP^HygR]) II* | Phenotyping assays | | 8 |
| APR867 | *lat-1 (apr9 [lat-1 (1-650)::eGFP^loxP^3xFLAG::lat-1 (651-1015) II* | Phenotyping assays | This paper | |
| APR965 | *lat-1(apr15[Δlat-1p(7kb)::lag-2p::lat-1 (1-650)::eGFP^loxP^3xFLAG::lat-1 (651-1015)]) II* | Phenotyping assays | This paper | |
| APR970 | *lat-1(apr16[Δlat-1p(7kb)::mex-5p::lat-1 (1-650)::eGFP^loxP^3xFLAG::lat-1 (651-1015)]) II* | Phenotyping assays | This paper | |

## Supplementary Table 5. Reliability and Reproducibility of the MD simulations

| **Reliability and reproducibility checklist for molecular dynamics simulations** | **Yes** | **No** | **Response (Please state where this information can be found in the text)** |
|---|---|---|---|
| **1. Convergence of simula6ons and analysis** | | | |
| 1a. Is an evaluation presented in the text to show that the property being measured has equilibrated in the simulations (*e.g.* time-course analysis)? | X | ☐ | Supplementary Fig. 5 shows such a time-course analysis. Note, the system is too big to become fully equilibrated within the simulation time. However, plotting the RMSD over simulation time shows that a plateau is reached in all three simulations, indicating that the system equilibrated at its local minimum. |
| 1b. Then, is it described in the text how simulations are split into equilibration and production runs and how much data were analyzed from production runs? | X | ☐ | Methods section: MD simulations |
| 1c. Are there at least 3 simulations per simulation condition with statistical analysis? | X | ☐ | Methods and results. |
| 1d. Is evidence provided in the text that the simulation results presented are independent of initial configuration? | X | | Simulations generally depend heavily on the starting model. Simulations were equilibrated independently and started after equilibration with different velocities, providing a certain level of independency. |
| **2. Connec6on to experiments** | | | |
| 2a. Are calculations provided that can connect to experiments (*e.g.* loss or gain in function from mutagenesis, binding assays, NMR chemical shifts, J-couplings, SAXS curves, interaction distances or FRET distances, structure factors, diffusion coefficients, bulk modulus and other mechanical properties, *etc.*)? | X | | Mutational analysis of predicted interface residues in the manuscript |
| **3. Method choice** | | | |
| 3a. Do simulations contain membranes, membrane proteins, intrinsically disordered proteins, glycans, nucleic acids, polymers, or cryptic ligand binding? | ☐ | X | Response not needed if **No** |

# Supplementary Methods

## Generation of *C. elegans* constructs

*hsp16.42p::lag-2(1-194)::VC155::lag-2(195-401)* (*VC::lag-2*, pNF17), *hsp16.42p::lag-2 (HA)* (*lag-2(HA)*, pSP403)

Plasmid pAW722 (kind gift of Alison Woollard) containing *hsp16.42p* followed by the C-terminal part of the split Venus (*VC155*) was modified by outward PCR using phosphorylated primers lat-1_1469Fp/lat-1_1470Rp, removing the C-terminal stop codon and introducing a 5x alanine linker as well as *Nco*I and *Not*I restriction sites (plasmid pNF1). Subsequently, the *lag-2* signal peptide sequence (annotated with SignalP 5.0) was introduced behind the *hsp16.42p* using primers lat-1_1483Fp/lat-1_1484Rp. *lag-2* without signal peptide was amplified from N2 genomic DNA using primers lat-1_1481F/lat-1_1482R and cloned into pCR2.1 using the Zero Blunt TOPO PCR Cloning Kit (Invitrogen). *lag-2(1-193)* was amplified using primers lat-1_1712F/lat-1_1713R, introducing *Xho*I sites, allowing for cloning upstream *VC155*. *lag-2(194-401)* was introduced into this vector by amplification of *lag-2* with primers lat-1_1714F/lat-1_1709R, introducing *Not*I sites. Subsequent digestion and ligation were performed, resulting in plasmid pNF17.

The FLAG tag of pNF17 was swapped with lat-1_2652F and lat-1_2653R that were phosphorylated prior to amplification, resulting in plasmid pSP403.

*hsp16.42p::VN173::lat-1* (*VN::lat-1*, pSP246), *hsp16.42p::VN173::lat-1 (V5)* (pSP404)

pAW721 (kind gift of Alison Woollard) containing *hsp16.42p* followed by the N-terminal part of the split Venus (VN173) was modified in two steps using primers Bifc_1F/Bifc_2R introducing a *Pme*I restriction site and primers Bifc_3Fp/Bifc_4Rp to remove the multiple cloning site and a Myc tag in the vector as well as to introduce *Eco*RI/*Xho*I sites (all by outward PCR). *lat-1* cDNA without signal peptide was amplified from pSP101 [1] with primers Bifc_5F/Bifc_6R, introducing *Pme*I sites. PCR product and backbone were digested and ligated. The *lat-1* signal peptide sequence (annotated with SignalP 5.0) was separately amplified (primers Bifc_7F/Bifc_8R) and ligated before VN173 via the previously added *Xho*I sites. C-terminal of *lat-1* signal peptide sequence, a FLAG tag was added using primers lat-1_1731Fp/lat-1_1732Rp (plasmid pSP188) together with a 3x GGGGS linker sequence (primers lat-1_2009Fp/lat-1_2006Rp), resulting in pSP246. Subsequently, the FLAG tag was exchanged for a V5 tag by outward PCR using phosphorylated lat-1_2654F/lat-1_2655R.

*hsp16.42p::VN173::lag-2* (*VN::lag-2*, pSP228)

In plasmid pNF1 (see above) *Xho*I/*Eco*RI sites were introduced by outward PCR with primers lat-1_1467Fp/lat-1_1468Rp (resulting in plasmid pNF2). The sequence of the *lag-2* signal peptide was added by outward PCR using primers lat-1_1483Fp/lat-1_1484Rp, followed by addition of a FLAG tag with primers lat-1_1694Fp/lat-1_1695Rp. *lag-2* without signal peptide was amplified from N2 genomic DNA and introduced into this construct using *Not*I restriction sites previously introduced by lat-1_1708F/lat-1_1709R. To exchange *VC155* for *VN173*, *VN173* was amplified with lat-1_1819F/lat-1_1852R from pSP188 while amplifying the backbone with lat-1_1853F/lat-1_1822R. *VC155* was subsequently removed using primers lat-1_1869Fp/lat-1_1870Rp using outward PCR.

*hsp16.42p::VC155::glp-1* (*VC::glp-1*, pSP229)

In plasmid pNF2 (see above) the sequence of the *glp-1* signal peptide was inserted using primers lat-1_1473Fp/lat-1_1474Rp. Subsequently, an HA tag was introduced with primers lat-

1_1694Fp/lat-1_1695Rp. *glp-1* was amplified from N2 genomic DNA using primers lat-1_1700F/lat-1_1703R and ligated into the plasmid, incorporating *Nco*I and *Not*I restriction sites.

*hsp16.42p::lag-2* (*lag-2*, pSP234)

*VN173*, 3x GGGGS linkers, and the FLAG tag were removed from pSP228 by outward PCR using the 5' phosphorylated primers lat-1_1927Fp/lat-1_1928Rp and the resulting product was religated resulting in pSP234.

*hsp16.42p::lat-1 (V5) RBL$^{mut}$ GAIN$^{mut}$ mutations* (*lat-1 RBL$^{mut}$* GAIN$^{mut}$, pSP406)

Five point mutations were introduced in the *lat-1* RBL domain based on the AlphaFold2 Multimer models (Fig. 3b-c) by outward PCR using phosphorylated primers lat-1_2619F/lat-1_2618R. Thereby, the respective nucleotides were changed to yield alanine residues.

The resulting construct was modified so that seven amino acids amino acids in the LAT-1 GAIN domain identified to be potentially involved in LAG-2 binding (Fig. 3c) were mutated to code for alanines. This was achieved by outward PCR using primers lat-1_2491F/lat-1_2492R, as well as lat-1_2511F/lat-1_2512R.

**Generation of cell culture constructs**

*HA::lat-1::FLAG* (*lat-1*, pSP334)

Codon harmonized *lat-1* cDNA (Genscript) was amplified without its signal peptide using primers lat-1_2169F/lat-1_2170R. In parallel, the backbone of pSP101 [1] containing an N-terminal HA tag followed by the mouse LPHN1 signal peptide, and a C-terminal FLAG tag was amplified using primers lat-1_2167F/lat-1_2168R. Both products were assembled using NEBuilder HiFi DNA Assembly Master Mix (New England Biolabs) according to manufacturer's instruction as described above, resulting in plasmid pSP360. *Kpn*I and *Eco*RI were used to excise this *lat-1* construct and ligate it into pcDNA5.1 (Thermo Fisher).

*HA::glp-1* (*glp-1*, pSP314)

Codon harmonized *glp-1* cDNA (Genscript) was inserted into pSP101 [1] employing the same strategy as for *lat-1* (pSP334), using primers lat-1_2175F/lat-1_2176R and lat-1_2177F/lat-1_2178R. The endogenous *glp-1* signal peptide (annotated with SignalP 5.0) was introduced by phosphorylated primers lat-1_2179F/lat-1_2180R performing a PCR on the previously generated plasmid, and ligation, resulting in plasmid pSP301. An N-terminal HA tag was introduced by outward PCR using phosphorylated primers lat-1_2240R/ lat-1_2241F.

*HA::lag-2* (*lag-2*, pSP340)

Codon harmonized *lag-2* cDNA (Genscript) with an N-terminal HA and a C-terminal FLAG tag (Genscript) was amplified with primers lat-1_2284F/lat-1_2285R, incorporating *Xma*I/*Kpn*I sites, respectively. Insertion into vector pcDps [2] was performed using restriction ligation as described above, resulting in plasmid pSP318. Subsequently, the FLAG tag was removed by outward PCR using phosphorylated primers lat-1_2402F/lat-1_2403R.

*Nluc::lag-2* (pSP359)

The nanoluciferase (Nluc) was amplified from previously generated plasmid pSP184 [3] using primers lat-1_2501F/lat-1_2502R. As backbone, pSP340 was amplified using primers lat-1_2499F/lat-1_2500R, incorporating a 20 bp sequence homology to the Nluc. The fragments were assembled using NEBuilder HiFi DNA Assembly Master Mix (New England Biolabs) according to manufacturer's instruction as described above, with the Nluc incorporated at the same place as VC155 in pSP403.

*Nluc::apx-1* (pSP427)

Plasmid pSP427 was synthesized by GenScript and cloned into pcDNA3.1. *Nluc* was inserted at the homologues site as for pSP359.

*Venus::lat-1* (pSP360)

As backbone, pSP334 was amplified using primers lat-1_2503F/lat-1_2504R, incorporating 20 bp overhangs to Venus. The Venus sequence was generated by PCR using primers lat-1_2505F/at-1_2506R. Both fragments were subsequently assembled using NEBuilder HiFi DNA Assembly Master Mix (New England Biolabs) according to manufacturer's instruction as described above.

*Venus::lat-1(RBLmut)* (pSP395)

For this construct, pSP360 served as backbone and the mutations leading to the change of the five residues within the RBL potentially involved in LAG-2 binding (Fig. 3c) to alanines were introduced using phosphorylated primers lat-1_2616R/lat-1_2617F and the resulting product was ligated.

*Venus::lat-1(GAIN$^{mut}$)* (pSP371)

In pSP360, mutations were introduced that result in seven amino acid point mutations (to alanines) within the GAIN domain identified to be potentially involved in LAG-2 binding (Fig. 3c). This was achieved by outward PCR of pSP360 with phosphorylated primes lat-1_2513F/lat-1_2514R and subsequent ligation.

*Venus::lat-1(RBL$^{mut}$ GAIN$^{mut}$)* (pSP392)

To generate a construct containing all point mutations, pSP371 was amplified using phosphorylated primers lat-1_2616F/ lat-1_2617R which introduce the RBL mutations. The resulting product was ligated.

*Venus::hGLP1R* (pSP396)

Human *GLP1R* was amplified from plasmid GLP1R-N-EGFP_pcDNA3.1 [3] using primers lat-1_2632F/lat-1_2633R. *HA::Venus* was amplified from pSP360 using primers lat-1_2630F/lat-1_2631R. As backbone, pcDNA3.1 was amplified using lat-1_2628F/lat-1_2629R. All three fragments assembled as described above using NEBuilder

HiFi DNA Assembly Master Mix (New England Biolabs) according to manufacturer's instruction.

### Genetic engineering of the *lat-1* locus

*lat-1(1-650)::eGFP::3xFLAG::lat-1(651-1015)* (strain APR867)

To edit the *lat-1* locus by CRISR-Cas9 genome editing, a self-excising cassette system where flanking sites corresponding to the endogenous *lat-1* as were incorporated as suggested [4]. Briefly, this construct was produced by amplification of a 629 bp 5'-flanking site as well as a 571 bp 3´-flanking site from plasmid pTL2 [6] using primers lat-1_2527F/lat-1_2528R and lat-1_2529F/lat-1_2530R, respectively. Flanking sites were incorporated into plasmid pJJR82 (gift from Mike Boxem, Addgene plasmid #75027) as instructed [5].

In parallel, an sgRNA targeting the *lat-1* locus was generated by cloning a suitable sgRNA (determined using Benchling [Biology Software]. (2024). Retrieved from https://benchling.com) into pJJR50 as previously described [5] using primers lat-1_2531F/lat-1_2532R (pSP373). pJJR50 was a gift from Mike Boxem (Addgene plasmid #75026).

Plasmids pSP372 and pSP373 were injected together in the syncytial germline of wild-type adult nematodes with *eft-3p::cas9-SV40_NLS::tbb-2 3'UTR* [6], pCFJ104, and pCFJ90 according to the instructions and at the concentrations indicated in [4]. Construct *eft-3p::cas9-SV40_NLS::tbb-2 3'UTR* was a gift from John Calarco (Addgene plasmid #46168 [6]). pCFJ104 and pCFJ90 were gifts from Erik Jorgensen (Addgene plasmids #19328 and #19327 [7]). Rolling, non-red F1 worms were recovered. The marker was removed using the embedded *hsp::*Cre recombinase, driven by a heat-shock promoter. Rolling L1/L2 animals were heat-shocked for 3 h and non-rolling offspring were recovered.

*lag-2p::lat-1(1-650)::eGFP::3xFLAG::lat-1(651-1015)* (strain APR965)

To modify the *lat-1* genomic locus to achieve tissue-specific expression in the DTC, 7 kb of the *lat-1* promoter were exchanged for the *lag-2* promoter (2 kb).

For this purpose, a plasmid containing *lag-2p* and *lat-1* flanking sites (pSP412) was generated. *lag-2p* was amplified from plasmid pJL1 [8] using primers lat-1_2695F/ lat-1_2696R. Primer pairs lat-1_2693F/lat-1_2694R and lat-1_2697F/lat-1_2698R were used to amplify 5' and 3' flanking sites (450 and 571 bp, respectively). pCR2.1 TOPO (Invitrogen) was used as backbone and was amplified using primers lat-1_2691F/lat-1_2692R. The resulting PCR products were assembled using NEBuilder HiFi DNA Assembly Master Mix (New England Biolabs) according to manufacturer's instruction. Subsequently, the *lag-2* promoter sequence and homology arms were amplified from this plasmid (pSP412) using primers lat-1_881F/lat-1_2036Rp. The phosphorylated strand of the PCR product was digested with λ-exonuclease, purified and injected together with RNPs containing crRNA 2 in adult nematodes of strain APR867 as described by [9]. Progeny was screened using the marker-free screening method as described in the same paper, resulting in strain APR934. The endogenous *lat-1p* was deleted using ssODN4 as repair template as well as crRNA 5 and 6 to cleave at the extremities of the anticipated promoter. This was performed as described by [10]. The resulting lines were outcrossed and homozygous lines isolated.

*mex-5p::lat-1(1-650)::eGFP::3xFLAG::lat-1(651-1015)* (strain APR970)

To modify the *lat-1* genomic locus to achieve tissue-specific expression of *lat-1* in the germ cells, 7 kb of the *lat-1* promoter were exchanged for the *mex-5* promoter (0.5 kb).

First, a construct containing the *mex-5p* and *lat-1* flanking sites (450 and 571 bp, respectively) was generated (pSP422). *mex-5p* was amplified from N2 genomic DNA using primers lat-1_2710F/lat-1_2711R. pSP412 was used as backbone, already containing the correct flanking sites, and was amplified using lat-1_2708F/lat-1_2709R. The resulting PCR products were assembled as described for pSP412. Subsequently, the promoter sequence and homology arms were amplified from plasmid pSP422 using primers lat-1_881F/lat-1_2036Rp, followed by the same procedure as described for *lag*-2p resulting in strain APR945.

The endogenous *lat-1p* was deleted using ssODN4 as repair template as well as crRNA 5 and 6 to cleave at the extremities of the anticipated promoter. This was performed as described by [10]. The resulting lines were outcrossed and homozygous lines isolated.

## Supplementary Capture Molecular Modeling

Generation of initial LAT-1 and LAG-2 interactions using AlphaFold2 Multimer

Structural models of the interaction interface between the two extracellular regions (ECR) were obtained through AlphaFold2 Multimer [18]. Respective sequences were downloaded from Uniprot [19] (LAT-1 of *C. elegans* G5EDW2, GLP-1 of *C. elegans* P13508, and LAG-2 of *C. elegans* P45442, accessed on 19 January 2023). Here, various combinations of domains were investigated, including:

- LAT-1 GAIN domain and full-length ECR of LAG-2
- LAT-1 GAIN domain + HRM and full-length ECR of LAG-2
- LAT-1 RBL domain and full-length ECR of LAG-2
- Full-length LAT-1 ECR with linker instead of HRM and full-length ECR of LAG-2
- Full-length ECR of LAT-1 and full-length ECR of LAG-2
- Full-length ECR of LAT-1 with investigated mutation in the GAIN domain and full-length ECR of LAG-2
- Full-length ECR of LAT-1 with investigated mutation in the GAIN domain and RBL domain, and full-length ECR of LAG-2
- Full-length ECR of GLP-1 and full-length ECR of LAT-1
- Short length ECR of GLP-1 (including residues 400-620, including EGF-like domain 9-10 and LNR 1-3) and full-length ECR of LAT-1

The first 30 N-terminal residues of LAT-1 were predicted to form an unstructured, flexible coil with no defined secondary structure. Such regions often introduce noise or variability in computational structure predictions and are unlikely to contribute directly to stable protein-protein interactions. Therefore, these residues were removed prior to modeling in order to improve model convergence and focus computational analysis on the structured, interaction-relevant domains of LAT-1.

After generating the various complexes, the models were visually inspected and common interaction sites highlighted. The best ranked models by AlphaFold2 Multimer internal scoring function was energetically minimized with the standard Rosetta3 energetical minimization (relax) function.

The workflow summarizes as follows:

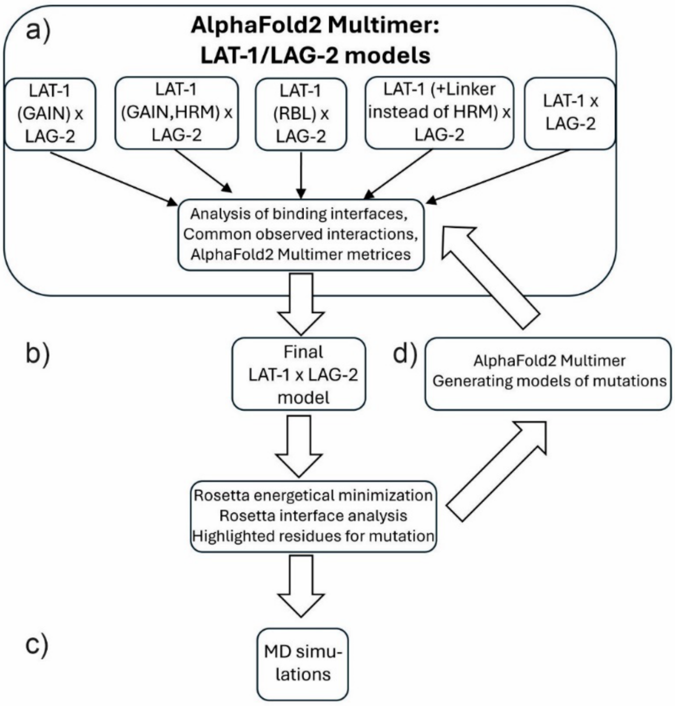

Protein sequences for various LAT-1 and LAG-2 complex were prepared and modeled using AlphaFold2 Multimer to predict initial complex structures (a)). The best-ranked models were assessed visually and computationally. A common binding interface between various models and combinations was highlighted. Based on the initial findings, the best structure from the entire LAT-1/LAG-2 complex was retrieved and structure prepared with the Rosetta Suite (b)). After energetical minimization, for the best-scoring models the interaction interface was analyzed using Rosetta InterfaceAnalyzer to estimate buried surface area, hydrogen bonding, and per-residue energetic contributions. Furthermore, promising residues in LAT-1 relevant for binding were highlighted for experimental analysis. To assess model quality, the structure was subject to molecular dynamics (MD) simulations (c)). Furthermore, AlphaFold2 Multimer was used again, to generate structural models of the proposed mutations (d)). These were analyzed within the established computational pipeline but without further quality validation with MD simulations. Structural visualizations were generated in PyMOL with domain annotations corresponding to functional regions of LAT-1 and LAG-2.

*<u>Sequence for LAT-1 and LAG-2 models</u>*

```
>LAT-1(https://www.uniprot.org/uniprotkb/G5EDW2/entry):
>N-ter
#first residues excluded as unstructured coil
MRRNKTTYSLLQTILVACLLTVTPTFASNK

PTTDESGTISHT

>RBL
ICDGEAAELSCPAGKVISIVLGNYGRFSVAVCLPDNDIVPSNINCQNHKTKSILEKKCNGDSMCYFTVDKKTFTE
DPCPNTPKYLEVKYNCV

>HRM
VPATTTTTTTTSTTTTDSSLIVDEEEEAQKDALNSDVIKPVKKKEDVFCSATNRRGVNWQNTKSGTTSSAPCPE
GSSGKQLWACTEEGQWLTEFPNSAGCESNWISSRNSVLSGVISSEDVSGLP

An additional linker was investigated instead of HRM
>linker
VPATTTGGGGGGGGGGGGGGGGGGGG

>GAIN
EFLRNLGSETRRPMVGGDLPKVLHLLEKTVNVIAEESWAYQHLPLSNKGAVEVMNYMLRNQEIWGSWDVTKRKEF
ASRFILAAEKAMVASAKGMMTSAESNVIVQPAITVEISHKIKMSSQPTDYILFPSAALWNGQNVDNVNIPRDAIL
KINKDETQVFFSSFDNLGAQMTPSDVTVAIAGTDQTEVRKRRVVSRIVGASLIENGKERRVENLTQPVRITFYHK
ESSVRHLSNPTCVWWNHHELKWKPSGCKLSYHNKTMTSCDCTHLTHFAVLMDVRGHDLNEIDQTLL

>Transmembrane area and intracellular region (deleted in the modeling step)
(TLLTYVGCIISIICLLLTFFAYLIFSRNGGDRVFIHENLCLSLAIAEITFLAGITRTEDSLQCGIIAVALMYMF
LSALTWMLLEGYHIHRMLTEVFPSDPRRFTYLLVGYIPPAIITLVAYLYNSDGFGTPDHCWLSTQNNFIWFFAGP
ACFIFCANSLVLVKTLCTVYQHTSGGYLPCRHDVDSGRSIRNWVKGSLALASLLGVTWIFGLFWVEDSRSIVMAY
VFTISNSLQGLFIFLFHVVFAEKMRKDVGHWMYRRGCGGSSNSSPNHKRHNVQRDLMSPGVNSSTGSDFLYNTND
KYLTNSDTTNRLVYNGIMNHPNQMSVYQQHPHHQIYEQQPGTYDYATIAYGDMMPGHRVAAPPAYQRLAVAEGRY
GSQHQLYQGWHHRPPPEFSPPPPPLSTGPPNSRHYGTGSSGRRPPSSKMSDDSAYSDGSSSMLTTEVTPQGQTVL
RIDLNKPSMYCQD)
```

in the refinement step, the tested mutations were introduced in AlphaFold Multimer, resulting in the two following LAT-1 sequence:

>LAT-1 refined (GAIN domain mutation)

PTTDESGTISHTICDGEAAELSCPAGKVISIVLGNYGRFSVAVCLPDNDIVPSNINCQNHKTKSILEKKCNGDSM
CYFTVDKKTFTEDPCPNTPKYLEVKYNCVVPATTTTTTTTTSTTTTDSSLIVDEEEEAQKDALNSDVIKPVKKKE
DVFCSATNRRGVNWQNTKSGTTSSAPCPEGSSGKQLWACTEEGQWLTEFPNSAGCESNWISSRNSVLSGVISSED
VSGLPEFLRNLGSETRRPMVGGDLPKVLHLLEKTVNVIAEESWAYQHLPLSNKGAVEVMNYMLRNQEIWGSWDVT
KRKEFASRFILAAEKAMVASAKGMMTSAESNVIVQPAITVEISHKIKMSS**AA**T**AA**I**A**FPSAA**AA**NGQNVDNVNIP
RDAILKINKDETQVFFSSFDNLGAQMTPSDVTVAIAGTDQTEVRKRRVVSRIVGASLIENGKERRVENLTQPVRI
TFYHKESSVRHLSNPTCVWWNHHELKWKPSGCKLSYHNKTMTSCDCTHLTHFAVLMDVRGHDLNEIDQTLL

>LAT-1 refined (GAIN domain and RBL domain mutation)

PTTDESGTISHTICDGEAAELSCPAGKVISIV**A**GNYGR**A**SVAVCLP**A**N**A**I**A**PSNINCQNHKTKSILEKKCNGDSM
CYFTVDKKTFTEDPCPNTPKYLEVKYNCVVPATTTTTTTTTSTTTTDSSLIVDEEEEAQKDALNSDVIKPVKKKE
DVFCSATNRRGVNWQNTKSGTTSSAPCPEGSSGKQLWACTEEGQWLTEFPNSAGCESNWISSRNSVLSGVISSED
VSGLPEFLRNLGSETRRPMVGGDLPKVLHLLEKTVNVIAEESWAYQHLPLSNKGAVEVMNYMLRNQEIWGSWDVT
KRKEFASRFILAAEKAMVASAKGMMTSAESNVIVQPAITVEISHKIKMSS**AA**T**AA**I**A**FPSAA**AA**NGQNVDNVNIP
RDAILKINKDETQVFFSSFDNLGAQMTPSDVTVAIAGTDQTEVRKRRVVSRIVGASLIENGKERRVENLTQPVRI
TFYHKESSVRHLSNPTCVWWNHHELKWKPSGCKLSYHNKTMTSCDCTHLTHFAVLMDVRGHDLNEIDQTLL

>LAG-2 https://www.uniprot.org/uniprotkb/P45442/entry

>N-ter

MIAYFLLLLTCLPVLQARVEVHQEFISSKRVSVRFEIVTESHSPNRPVTFDLFPRGPKTNIILLDTFNPVFNFSI
QLVQPFTGQPLGDRIYRKVQFSGTNQPWINDTFTTTSGISLSVATE

>DSL

VTCARNYFGNRCENFCDAHLAKAARKRCDAMGRLRCDIGWMGPHC

>EGF

GQAVDPRKCSCENDGICVSSMIHPSQPNQTSSNEQLICECTNGFTGTRCEIFGFNQFQLTAP

>EGF

RPDACSVKDACLNGAKCFPNGPKVFCSCAVGFIGEFCEISLTTTTP

>Transmembrane area (deleted in the modeling step)

TTVEITVSTSGYSSAVYITVALFVIFSIIIGCFKYKFKPMRQQALARGQVPEPYKMPETKSMLIDPEA
SEAQKKVFTIEGSVQKIDEEVRYTSAPRKYESNNEYAVIQKSTPPPPSLSPPSIPACHYV

*Sequence for LAG-2 and GLP-1 models*

>GLP-1(https://www.uniprot.org/uniprotkb/P13508/entry):

>N-ter
MRVLLILLAFFAPIASQLMGGECGREGACSVNGKCYNGKLIETYWCRCKKGFGGAFCERECDLDCKRG
EKCIYDVYGENPTCICQDCEDETPPTERTQKGCEEGYGGPDCKTPLFSGVNPCDSDPCNNGLCYPFYG
GFQCICNNGYGGSYCEEGIDHCAQNECAEGSTCVNSVYNYYCDCPIGKSGRYCERTECALMGNICNHG
RCIPNRDEDKNFRCVCDSGYEGEFCNKDKNECLIEETCVNNSTCFNLHGDFTCTCKPGYAGKYCEEAI
DMCKDYVCQNDGYCAHDSNQMPICYCEQGFTGQRCEIECPSGFGGIHCDLPLQRPHCSRSNGTCYNDG
RCINGFCVCEPDYIGDRCEINRKDFKFPDIQSCKYNPCVNNATCIDLKNSGYSCHCPLGFYGLNCEQH
LLCTPTTCANGGTCEGVNGVIRCNCPNGFSGDYCEIKDRQLCSRHPCKNGGVCKNTGYCECQYGYTGP
TCEEVLVIEKSKETVIRDLCEQRKCMDLASNGICNPECNLEECNFDGGDCSGGQRPFSKCQYPARCAD

QFANGVCNQECNNEECLYDGLDCQSELFRCPAHIRKHCIERRGDGVCNLECSFIGCGFDGGDCNNGTE
AIILSDIRIKVQIDPIEFQ

>Transmembrane area and intracellular region (deleted in the modeling step)

ATGGETLMQISANLRATVRIQRDELGPLVFRWDGEHEMERVEMNSSKLEDQFVLSHHVRRYRQAVVTGIVLYLEV
EEICKPEFCRFSTAQSVVDLIAAGLVKSDGRMSLGLPITEAMVAVPKRNEIDEGWSRSQVILFACIAFLAFGTVV
AGVIAKNGPERSRKRKMVNATVWMPPMESTNEKGRRNQSNHSSQCSLLDNSAYYHPNTKRHCSDYSTGYNGEQYS
QIYPQTLANGYPGDYNELNFDFQSETFAPADLPADEIPLHVQAAGPDAITAPITNESVNQVDSKYRRRVLHWLAA
NVRGKPEDVITTEAIRCLKAGADVNARDCDENTALMLAVRAHRVRLSVVLLREGANPTIFNNSERSALHEAVVNK
DLRILRHLLTDKRLLKEIDELDRNGMTALMLVARELGKHQVEMAELLLSKGAKLDYDGAARKDSNKYKGRTALHY
AAMHDNEEMVIMLVRRSSNKDKQDEDGRTPIMLAAKEGCEKTVQYLALNDASLGIVDSMDMTAAQVAEASYHHEL
AAFLRQVANERHRNDIMRQQIVKSGHGAKSGRQTVKNIKRAGSRKTPTSAASSRETNHLTPPPSDGSFSSPSPHY
YPTTTSTPNRMETSPEYMFNHEMAPPVNAMWYTTPPPYQDPNYRHVPPNTAFQNAEQMNGSFYC

>shorten to res 400-620 including EGF-like domain 9-10 and LNR1-3

GFYGLNCEQHLLCTPTTCANGGTCEGVNGVIRCNCPNGFSGDYCEIKDRQLCSRHPCKNGGVCKNTGYCECQYGY
TGPTCEEVLVIEKSKETVIRDLCEQRKCMDLASNGICNPECNLEECNFDGGDCSGGQRPFSKCQYPARCADQFAN
GVCNQECNNEECLYDGLDCQSELFRCPAHIRKHCIERRGDGVCNLECSFIGCGFDGGDCNNGTEAIILSDIRIKV
QIDPIEFQATGGETLMQISANLRATVRIQRDELGPLVFRWDGEHEMERVEMNSSKLEDQFVLSHHVRRYRQAVVT
GIVLYLEVEEICKPEFCRFSTAQSVVDLIAAGLVKSDGRMSLGLPITEAMVAVPKRNEIDEG

*__Sequence for LAT-1 models__*

*__AlphaFold Multimer SLURM script at Leipzig Rechenzentrum__*

```
--------------------------------
#!/bin/bash
#SBATCH --job-name=alphafold-test
#SBATCH --output=alphafold-test-job-out.%J
#SBATCH --time=12:00:00
#SBATCH --nodes=1
#SBATCH --ntasks-per-node=16
#SBATCH --gres=gpu:a30:1
#SBATCH --partition=paula

module purge
ml AlphaFold/2.2.2-foss-2021b-CUDA-11.4.1

export ALPHAFOLD_DATA_DIR=/software/databases/alphafold
export ALPHAFOLD_HHBLITS_N_CPU=12

alphafold --fasta_paths=/home/sc.uni-
leipzig.de/<user>/LAT1_LAG2/just_GAIN.fasta --max_template_date=2100-05-14
--data_dir=/software/databases/alphafold --output_dir=/home/sc.uni-
leipzig.de/<user>/LAT1_LAG2/alphafold_output --db_preset=full_dbs  --
model_preset=multimer  --use_gpu_relax=True  --
num_multimer_predictions_per_model=10  --
pdb_seqres_database_path=/software/databases/alphafold/pdb_seqres/pdb_seqre
s.txt

--------------------------------
```

###input example for GAIN domain of LAT-1 and full ECR of LAG-2

>LAG2

```
MIAYFLLLLTCLPVLQARVEVHQEFISSKRVSVRFEIVTESHSPNRPVTFDLFPRGPKTNIILLDTFNPVFNFSI
QLVQPFTGQPLGDRIYRKVQFSGTNQPWINDTFTTTSGISLSVATVTFDLFPRGPKTNIILLDTFNPVFNFSIQL
VQPFTGQPLGDRIYRKVQFSGTNQPWINDTFTTTSGISLSVATVTCARNYFGNRCENFCDAHLAKAARKRCDAMG
RLRCDIGWMGPHCGQAVDPRKCSCENDGICVSSMIHPSQPNQTSSNEQLICECTNGFTGTRCEIFGFNQFQLTAP
RPDACSVKDACLNGAKCFPNGPKVFCSCAVGFIGEFCEISLTTTTP
```

```
>GAIN
```

```
NWISSRNSVLSGVISSEDVSGLPEFLRNLGSETRRPMVGGDLPKVLHLLEKTVNVIAEESWAYQHLPLSNKGAVE
VMNYMLRNQEIWGSWDVTKRKEFASRFILAAEKAMVASAKGMMTSAESNVIVQPAITVEISHKIKMSSQPTDYIL
FPSAALWNGQNVDNVNIPRDAILKINKDETQVFFSSFDNLGAQMTPSDVTVAIAGTDQTEVRKRRVVSRIVGASL
IENGKERRVENLTQPVRITFYHKESSVRHLSNPTCVWWNHHELKWKPSGCKLSYHNKTMTSCDCTHLTHFAVLMD
VRGHDLNEIDQTLL
```

*__Options for Energy Minimization (300 generated structures in 30 runs)__*

```
/$Rosetta/main/source/bin/relax.static.linuxggcrelease @flags.options -s
<selected_model>.pdb
-------------------------------
#flags.options
# i/o
-out:pdb
-out:path:all output/
-nstruct 10

# relax options
-default_max_cycles 200
-relax:min_type lbfgs_armijo_nonmonotone
-relax:constrain_relax_to_start_coords

# reduce memory footprint
-chemical:exclude_patches LowerDNA  UpperDNA Cterm_amidation SpecialRotamer
VirtualBB ShoveBB VirtualDNAPhosphate VirtualNTerm CTermConnect sc_orbitals
pro_hydroxylated_case1 pro_hydroxylated_case2 ser_phosphorylated
thr_phosphorylated  tyr_phosphorylated tyr_sulfated lys_dimethylated
lys_monomethylated  lys_trimethylated lys_acetylated glu_carboxylated
cys_acetylated tyr_diiodinated N_acetylated C_methylamidated
MethylatedProteinCterm

-linmem_ig 10

# run multiple processes to produce output for one file
-multiple_processes_writing_to_one_directory
-------------------------------
```

Based on the built structures, the RMSD to the respective best model for each model generation was calculated, a *rmsd-total_score* plot generated, and the structures investigated. It must be noted that most structures had an RMSD-value of below 2 Å and clustering was omitted.

An additional interface analyzer for all energetically minimized structures was run to highlight the best interactions.

```
-------------------------------
```

```
/$Rosetta/main/source/bin/rosetta_scripts.static.linuxggcrelease -
parser:protocol InterfaceAnalyzer.xml -s <selected_model>.pdb
-------------------------------
#InterfaceAnalyzer.xml
<ROSETTASCRIPTS>
    <TASKOPERATIONS>
    </TASKOPERATIONS>
    <SCOREFXNS>
        <ScoreFunction name="r15" weights="ref2015" symmetric="0" />
    </SCOREFXNS>
    <FILTERS>
    </FILTERS>
    <RESIDUE_SELECTORS>
    </RESIDUE_SELECTORS>
    <MOVERS>
        <InterfaceAnalyzerMover name="Ana" scorefxn="r15"
pack_separated="false" pack_input="false" resfile="false" packstat="false"
interface_sc="false" tracer="false" use_jobname="false" interface="A_B" />
    </MOVERS>
    <APPLY_TO_POSE>
    </APPLY_TO_POSE>
    <PROTOCOLS>
        <Add mover_name="Ana"/>
    </PROTOCOLS>
</ROSETTASCRIPTS>
-------------------------------
```

*<u>Energetical investigation and energy breakdown</u>*

The best docked models were selected for an energetical hotspot analysis with the Rosetta *per_residue_energies* application:

```
-------------------------------
/$Rosetta/main/source/bin/residue_energy_breakdown.default.linuxgccreleas
e -s *.pdb -out:file:silent data.out
-------------------------------
```

A following python script was utilized to investigate the output and select the per residue energy contributions relevant for the peptide and generating a contact map:

```
-------------------------------
import pandas as pd
import seaborn as sns
import matplotlib.pyplot as plt
import numpy as np

from datetime import date

df1 = pd.read_csv('data.out', sep='\s+')

df2 = df1.loc[(df1['resi2'] != '--')]
df2['resi1'] = df2['resi1'].astype(int)
df2['resi2'] = df2['resi2'].astype(int)
df3 = df2.loc[(df2['resi1'] < 287) & (df2['resi2'] > 286)]
df3.to_csv('selected_ia_h.list', sep='\t', index=None)
df3.to_csv('selected_ia.list', sep='\t', index=None, header=None)
peptide_res = df3['resi2'].to_list()
peptide_res = list(set(peptide_res))
```

```
TM_res = df3['resi1'].to_list()
TM_res = list(set(TM_res))
filler = []
for lines in open('selected_ia.list', 'r').readlines():
    line = lines.split('\t')
    filler.append(line)
fil = open('energy_ia.list', 'w')
for TM in TM_res:
    for peptide in peptide_res:
        counter = 0
        for ele in filler:
            if int(ele[5]) == peptide and int(ele[2]) == TM:
                counter += float(ele[27])
        print(TM, peptide, counter, counter/1)
        print(TM, peptide, counter, counter/1, file=fil)
fil.close()

fil = open('summed_energy.list' ,'w')
final_energy = []
energy_ia = pd.read_csv('energy_ia.list', sep=' ', header=None)
for TM in TM_res:
    filler = energy_ia.loc[energy_ia[0] == TM]
    print(TM, filler[3].sum(), file=fil)
    final_energy.append((TM, filler[3].sum()))
fil.close()
filler = []
for lines in open('selected_ia.list', 'r').readlines():
    line = lines.split('\t')
    filler.append((line[5], line[7]))
    filler.append((line[2], line[4]))
filler = list(set(filler))

my_dict = {key: value for key, value in filler}
print(my_dict)
fil = open('energy_header.list', 'w')
for lines in open('energy_ia.list', 'r').readlines():
    line = lines.split()
    print(line[0] + "_" + my_dict[line[0]], line[1] + "_" +
my_dict[line[1]], line[2], line[3], file=fil)
fil.close()

df = pd.read_csv('energy_header.list', sep='\s+', header=None)
df = df.sort_values(by=[0])
# pivot the dataframe to create the desired format
df_pivoted = df.pivot(index=0, columns=1, values=3)
df_pivoted = df_pivoted.sort_index()
df_pivoted.to_csv('final_matrix.list', sep='\t')

#Heat Map
df = df_pivoted
fig, ax = plt.subplots(figsize=(6,8))
mask_con_corr = df[(df >= 0.1) | (df <= -0.1)]
label = df.columns.values.tolist()
index = df.index.tolist()

heat_map = sns.heatmap(mask_con_corr,
                       linewidths=1,linecolor='black',
                       #center=0,
                       cmap="BuPu_r",
                       cbar=True,
                       yticklabels=index, xticklabels=label,
```

```
vmin=-3, vmax=1)#, annot=True, fmt='g')
ax.xaxis.tick_top()
plottitle = "Energetical Analysis of Hotspot Residues in the \n
LAT-1/LAG-2 Interface"
plt.title(plottitle, fontsize=12)
plt.xticks(size=8)
plt.yticks(size=8)
heat_map.set_xticklabels(heat_map.get_xticklabels(), rotation=30)
heat_map.set_yticklabels(heat_map.get_yticklabels(), rotation=30)

ax.tick_params(left=False, bottom=False)
fig.savefig("_matrix_interaction.png", dpi=600)
-------------------------------
```

## Supplementary References


1. Müller, A. *et al.* Oriented Cell Division in the C. elegans Embryo Is Coordinated by G-Protein Signaling Dependent on the Adhesion GPCR LAT-1. *PLoS Genet.* **11**, e1005624 (2015).
2. Okayama, H. & Berg, P. A cDNA cloning vector that permits expression of cDNA inserts in mammalian cells. *Mol. Cell. Biol.* **3**, 280–289 (1983).
3. Wygas, M. M., Laugwitz, J. M., Schmidt, P., Elgeti, M. & Kaiser, A. Dynamics of the Second Extracellular Loop Control Transducer Coupling of Peptide-Activated GPCRs. *Int. J. Mol. Sci.* **24**, (2023).
4. Dickinson, D. J., Pani, A. M., Heppert, J. K., Higgins, C. D. & Goldstein, B. Streamlined Genome Engineering with a Self-Excising Drug Selection Cassette. *Genetics* **200**, 1035–1049 (2015).
5. Waaijers, S. *et al.* A tissue-specific protein purification approach in Caenorhabditis elegans identifies novel interaction partners of DLG-1/Discs large. *BMC Biol.* **14**, 66 (2016).
6. Friedland, A. E. *et al.* Heritable genome editing in C. elegans via a CRISPR-Cas9 system. *Nat. Methods* **10**, 741–743 (2013).
7. Frøkjaer-Jensen, C. *et al.* Single-copy insertion of transgenes in Caenorhabditis elegans. *Nat. Genet.* **40**, 1375–1383 (2008).
8. Matúš, D. *et al.* The N terminus-only (trans) function of the Adhesion GPCR Latrophilin-1 controls multiple processes in reproduction of C. elegans. *G3 Bethesda Md* (2024) doi:10.1093/g3journal/jkae206.
9. Eroglu, M., Yu, B. & Derry, W. B. Efficient CRISPR/Cas9 mediated large insertions using long single-stranded oligonucleotide donors in C. elegans. *FEBS J.* **290**, 4429–4439 (2023).
10. Ghanta, K. S., Ishidate, T. & Mello, C. C. Microinjection for precision genome editing in Caenorhabditis elegans. *STAR Protoc.* **2**, 100748 (2021).
11. Brenner, S. The genetics of Caenorhabditis elegans. *Genetics* **77**, 71–94 (1974).
12. Voronina, E., Paix, A. & Seydoux, G. The P granule component PGL-1 promotes the localization and silencing activity of the PUF protein FBF-2 in germline stem cells. *Dev. Camb. Engl.* **139**, 3732–3740 (2012).
13. Henderson, S. T., Gao, D., Lambie, E. J. & Kimble, J. lag-2 may encode a signaling ligand for the GLP-1 and LIN-12 receptors of C. elegans. *Dev. Camb. Engl.* **120**, 2913–2924 (1994).
14. Komatsu, H. *et al.* OSM-11 Facilitates LIN-12 Notch Signaling during Caenorhabditis elegans Vulval Development. *PLoS Biol.* **6**, e196 (2008).
15. Kodoyianni, V., Maine, E. M. & Kimble, J. Molecular basis of loss-of-function mutations in the glp-1 gene of Caenorhabditis elegans. *Mol. Biol. Cell* **3**, 1199–1213 (1992).
16. Sorensen, E. B., Seidel, H. S., Crittenden, S. L., Ballard, J. H. & Kimble, J. A toolkit of tagged glp-1 alleles reveals strong glp-1 expression in the germline, embryo, and spermatheca. *MicroPublication Biol.* **2020**, (2020).
17. Byrd, D. T., Knobel, K., Affeldt, K., Crittenden, S. L. & Kimble, J. A DTC niche plexus surrounds the germline stem cell pool in Caenorhabditis elegans. *PloS One* **9**, e88372 (2014).
18. Evans, R. *et al. Protein Complex Prediction with AlphaFold-Multimer*. (2021). doi:10.1101/2021.10.04.463034.
19. Zaru, R. & Orchard, S. UniProt Tools: BLAST, Align, Peptide Search, and ID Mapping. *Curr. Protoc.* **3**, e697 (2023).
