# Supplementary material for: Notch activity is modulated by the aGPCR Latrophilin binding the DSL ligand in C. elegans
Source: Nat Commun. 2025 Jul 12;16:6461. doi: 10.1038/s41467-025-61730-0 (PMC12255766; doi:10.1038/s41467-025-61730-0)
Supplement: Supplementary file 2 — Reporting Summary [file 41467_2025_61730_MOESM2_ESM.pdf]

nature portfolio

Corresponding author(s): Simone Prömel
Last updated by author(s): Jun 26, 2025

Reporting Summary

Nature Portfolio wishes to improve the reproducibility of the work that we publish. This form provides structure for consistency and transparency in reporting. For further information on Nature Portfolio policies, see our Editorial Policies and the Editorial Policy Checklist.

Statistics

For all statistical analyses, confirm that the following items are present in the figure legend, table legend, main text, or Methods section.

n/a	Confirmed	
☐	☒	The exact sample size (*n*) for each experimental group/condition, given as a discrete number and unit of measurement
☐	☒	A statement on whether measurements were taken from distinct samples or whether the same sample was measured repeatedly
☐	☒	The statistical test(s) used AND whether they are one- or two-sided<br>*Only common tests should be described solely by name; describe more complex techniques in the Methods section.*
☐	☒	A description of all covariates tested
☐	☒	A description of any assumptions or corrections, such as tests of normality and adjustment for multiple comparisons
☐	☒	A full description of the statistical parameters including central tendency (e.g. means) or other basic estimates (e.g. regression coefficient) AND variation (e.g. standard deviation) or associated estimates of uncertainty (e.g. confidence intervals)
☐	☒	For null hypothesis testing, the test statistic (e.g. *F*, *t*, *r*) with confidence intervals, effect sizes, degrees of freedom and *P* value noted<br>*Give P values as exact values whenever suitable.*
☒	☐	For Bayesian analysis, information on the choice of priors and Markov chain Monte Carlo settings
☒	☐	For hierarchical and complex designs, identification of the appropriate level for tests and full reporting of outcomes
☒	☐	Estimates of effect sizes (e.g. Cohen's *d*, Pearson's *r*), indicating how they were calculated

Our web collection on statistics for biologists contains articles on many of the points above.

Software and code

Policy information about availability of computer code

Data collection	AlphaFold2 Multimer, Python, Rosetta, CHARMM36 forcefield for GROMACS (v. 2024.2)
Data analysis	PyMOL, GraphPad PRISM, Python, Rosetta, CHARMM36 forcefield for GROMACS (v. 2024.2)

For manuscripts utilizing custom algorithms or software that are central to the research but not yet described in published literature, software must be made available to editors and reviewers. We strongly encourage code deposition in a community repository (e.g. GitHub). See the Nature Portfolio guidelines for submitting code & software for further information.

Data

Policy information about availability of data

All manuscripts must include a data availability statement. This statement should provide the following information, where applicable:

- Accession codes, unique identifiers, or web links for publicly available datasets
- A description of any restrictions on data availability
- For clinical datasets or third party data, please ensure that the statement adheres to our policy

Source data and supplementary information are provided with this paper. All other data and worm strains generated in this study are available upon request to the corresponding author.

Research involving human participants, their data, or biological material

Policy information about studies with human participants or human data. See also policy information about sex, gender (identity/presentation), and sexual orientation and race, ethnicity and racism.

Reporting on sex and gender	No human participants or data were used in the study.
Reporting on race, ethnicity, or other socially relevant groupings	No human participants or data were used in the study.
Population characteristics	No human participants or data were used in the study.
Recruitment	No human participants or data were used in the study.
Ethics oversight	No human participants or data were used in the study.

Note that full information on the approval of the study protocol must also be provided in the manuscript.

Field-specific reporting

Please select the one below that is the best fit for your research. If you are not sure, read the appropriate sections before making your selection.

[x] Life sciences [ ] Behavioural & social sciences [ ] Ecological, evolutionary & environmental sciences

For a reference copy of the document with all sections, see nature.com/documents/nr-reporting-summary-flat.pdf

Life sciences study design

All studies must disclose on these points even when the disclosure is negative.

Sample size	No sample-size calculation was performed. Sizes of C. elegans samples are usually larger than n = 10 within one group, providing the basis for solid statistical evalulation.
Data exclusions	For C. elegans phenotype analyses shown in Figs. 1, 2, 4, and 5 as well as in Supplementary Fig. 1, data of 1-3 entire assays were excluded, mainly because of temperature fluctuations.
Replication	All data were replicated at least three times for statistical analyses. Expression of constructs by Western blot analyses was qualitatively assessed and performed twice. Replications were successful and showed reproducibility of the data.
Randomization	The allocation of samples to the groups was random (e.g. C. elegans individuals of a certain genotype were randomly selected.
Blinding	Investigators were blinded to group allocations for all C. elegans experiments except expression analyses. This approach was also not possible for cell culture analyses.

Reporting for specific materials, systems and methods

We require information from authors about some types of materials, experimental systems and methods used in many studies. Here, indicate whether each material, system or method listed is relevant to your study. If you are not sure if a list item applies to your research, read the appropriate section before selecting a response.

Materials & experimental systems

n/a	Involved in the study	
[ ]	[x]	Antibodies
[ ]	[x]	Eukaryotic cell lines
[x]	[ ]	Palaeontology and archaeology
[ ]	[x]	Animals and other organisms
[x]	[ ]	Clinical data
[x]	[ ]	Dual use research of concern
[x]	[ ]	Plants

Methods

n/a	Involved in the study	
[x]	[ ]	ChIP-seq
[x]	[ ]	Flow cytometry
[x]	[ ]	MRI-based neuroimaging

Antibodies

Antibodies used	Rabbit anti-phospho Histone H3 (Ser10), Merck Millipore, 06-570<br>Mouse anti-V5 SV5-Pk1, BioRad, MCA1360

Anti-HA-Peroxidase, High Affinity, Sigma, 54193500
Goat anti-Rabbit IRDye 680RD-conjugated, LiCor, 926-68071
Goat anti-Mouse IgG (H+L), F(ab')2 Fragment CF 568, Biotium, 20109
Anti-rabbit IgG, HRP-linked Antibody, Cell Signaling Technology, 7074
Goat Anti-Mouse IgG (H+L)-HRP Conjugate, BioRad, 1721011
anti-Actin hFAB-Rhodamine, BioRad, 12004164

Validation

All primary antibodies were published previously (by us or other groups) as specific antibodies for their relevant targets.

Eukaryotic cell lines

Policy information about cell lines and Sex and Gender in Research

Cell line source(s)

HEK293 and HEK293T, acquired from German Collection of Microorganisms and Cell Cultures (DSMZ) (idenifier ACC 305 and ACC635, respectively)

Authentication

No authentication was performed by the authors as the cells were obtained from German Collection of Microorganisms and Cell Cultures (DSMZ) .

Mycoplasma contamination

All cell lines tested negative for mycoplasma contamination.

Commonly misidentified lines
(See ICLAC register)

No commonly misidentified cell lines were used in this study.

Animals and other research organisms

Policy information about studies involving animals; ARRIVE guidelines recommended for reporting animal research, and Sex and Gender in Research

Laboratory animals

Caenorhabditis elegans
Wild-type strain N2
other strains: APR873, WD881, APR886, JH2874, APR756, JK1277, APR569, HA1019, DG2389, APR768, JK5933, APR778, APR868, APR862, JK4475, APR524, APR739, APR751, APR771, APR834, APR841, APR842, APR920, APR974, APR742, APR867, APR965, APR970

Wild animals

The study did not involve wild animals.

Reporting on sex

Solely hermaphrodites were used for experiments in this study. Male individuals were solely employed for genetic crosses.

Field-collected samples

The study did not contain samples collected in the field.

Ethics oversight

Ethical approvel etc. is not required for the work with C. elegans.

Note that full information on the approval of the study protocol must also be provided in the manuscript.

Plants

Seed stocks

No plants were used in the study.

Novel plant genotypes

No plants were used in the study.

Authentication

No plants were used in the study.
